# Genome-wide Identification and Characterization of Heat Shock Protein Family Reveals Role in Development and Stress Conditions in *Triticum aestivum* L.

**DOI:** 10.1038/s41598-020-64746-2

**Published:** 2020-05-12

**Authors:** Ashish Kumar, Saloni Sharma, Venkatesh Chunduri, Amandeep Kaur, Satinder Kaur, Nikhil Malhotra, Aman Kumar, Payal Kapoor, Anita Kumari, Jaspreet Kaur, Humira Sonah, Monika Garg

**Affiliations:** 1 South Asian University, Chankyapuri, New Delhi, 110021 India; 2 Agri-Biotechnology Division, National Agri-Food Biotechnology Institute (NABI), S.A.S. Nagar (Mohali), Punjab India; 3 Punjab Agricultural University, Ludhiana, 141004 India; 4 UIET, PU, Chandigarh, 160014 India

**Keywords:** Expression systems, Gene ontology

## Abstract

Heat shock proteins (HSPs) have a significant role in protein folding and are considered as prominent candidates for development of heat-tolerant crops. Understanding of wheat HSPs has great importance since wheat is severely affected by heat stress, particularly during the grain filling stage. In the present study, efforts were made to identify HSPs in wheat and to understand their role during plant development and under different stress conditions. HSPs in wheat genome were first identified by using Position-Specific Scoring Matrix (PSSMs) of known HSP domains and then also confirmed by sequence homology with already known HSPs. Collectively, 753 *TaHSPs* including 169 *TaSHSP*, 273 *TaHSP40*, 95 *TaHSP60*, 114 *TaHSP70*, 18 *TaHSP90* and 84 *TaHSP100* were identified in the wheat genome. Compared with other grass species, number of HSPs in wheat was relatively high probably due to the higher ploidy level. Large number of tandem duplication was identified in *TaHSP*s, especially *TaSHSP*s. The *TaHSP* genes showed random distribution on chromosomes, however, there were more *TaHSP*s in B and D sub-genomes as compared to the A sub-genome. Extensive computational analysis was performed using the available genomic resources to understand gene structure, gene expression and phylogentic relationship of *TaHSPs*. Interestingly, apart from high expression under heat stress, high expression of *TaSHSP* was also observed during seed development. The study provided a list of candidate HSP genes for improving thermo tolerance during developmental stages and also for understanding the seed development process in bread wheat.

## Introduction

The sessile nature of plants makes them vulnerable to various kinds of biotic and abiotic stresses. Plants have sophisticated mechanisms to recognize and respond to these stresses. High-temperature stress is common abiotic stress, which significantly reduces the crop yield world-wide. Simulation modeling has predicted that every 1 °C rise in temperature above 30 °C reduces the grain filling duration by 0.30–0.60% and grain yield by 1.0–1.6%^1^. In response to high-temperature stress, plants synthesize many stress-responsive proteins including a family of proteins called heat shock proteins (HSPs). Enhanced production of HSPs has also been reported under other stress conditions like salinity, heavy metal, and drought^2,3^. Some HSPs are also involved in the development of viral infections too, in both plants and animals^4,5^. HSPs function as chaperones and assist in the refolding of denatured proteins, folding of nascent polypeptides and resolubilization of denatured protein aggregates^6,7^. With increasing concerns about global warming and climate change, many laboratories across the world have used HSPs to create thermo-tolerant plants^8,9^. There are reports describing the direct or indirect involvement of HSPs in different developmental stages of the plant^10^. Various HSPs show tissue-specific and developmental stage specific expression, such as some HSP70s which are not detectable in flower and young silique but are present at high concentrations in dry seed and their expression levels decrease upon germination^11^. Accumulation of HSPs, especially SHSPs has been observed during seed development in wheat by proteomic analysis^12,13^. The wide variety of roles played by HSPs in plants makes it very important to study them comprehensively.

HSPs are classified based on of their molecular weight, which ranges from 10 to 200 kDa. There are six major sub-families of heat shock proteins viz., HSP100 (ClpB), HSP90, HSP70/DnaK, HSP60 (GroEL/GroES), HSP40 (DnaJ) and small HSP (SHSP). Genome-wide characterization of HSP family has been performed in Soyabean (*Glycine max*)^14^, *Arabidopsis*^11^, Foxtail millet (*Setaria italica*)^15^, and Rice (*Oryza sativa*)^16^ (Table 1). However, in wheat, very limited information is available about HSP family in spite of it being the most important food crop which is severely affected by high temperature stress in several ways, especially during grain filling^17^. Apart from a recent report where characterization of only the SHSP sub-family was reported^2^, there is no significant study addressing HSPs in wheat. The recent availability of high-quality annotated reference genome by the International Wheat Genome Sequencing Consortium has facilitated the identification of HSP gene family^18^. Efforts were also made to identify potential HSP candidates, which showed significant changes in gene expression levels during stress or different developmental stages. In the present study, a comprehensive analysis of wheat HSP family was done via detailed bioinformatics analysis. This endeavor will be helpful to understand the role of HSPs under stress conditions in plants and their sequential implementation to increase thermo-tolerance in wheat.

**Table 1:** Number of HSPs in different plant species.

Plant sp. (ploidy)	Genome size (approx.)	Coding genes	SHSP	HSP40	HSP60	HSP70	HSP90	HSP100
***T. aestivum*** **(6n)**	17 Gb	107891	169	273	95	114	18	84
***S. italica*** **(2n)**	490 Mb	35831	37	—	20	27	9	20
***O. sativa*** **(2n)**	500 Mb	37960	12	—	3	6	3	3
***A. thaliana*** **(2n)**	135 Mb	27655	19	—	16	18	7	4

## Results and Discussion

### HSP gene identification, chromosome mapping, gene duplication and domain organization

**Figure 1:**
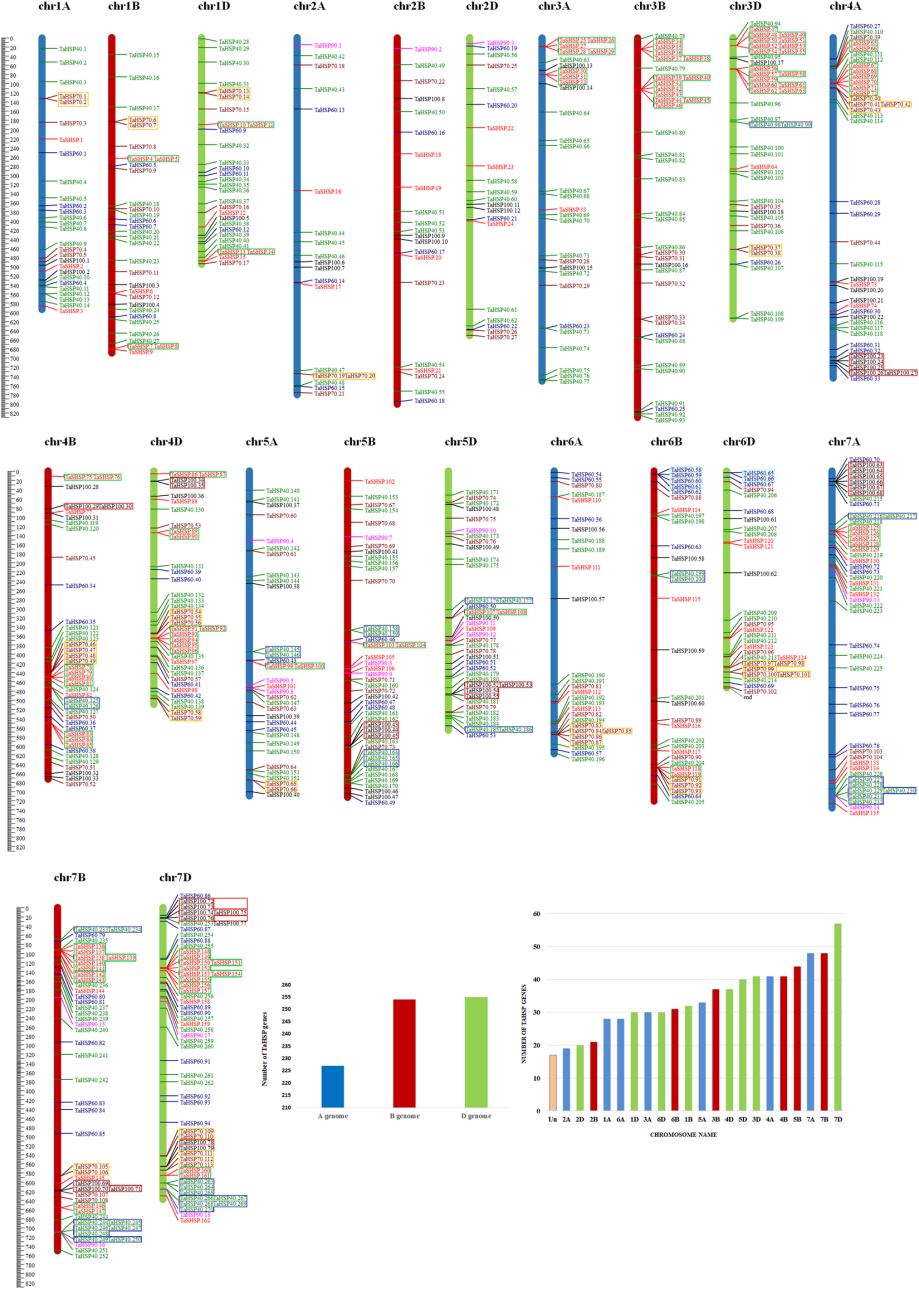
Distribution of HSP genes on the 21 chromosomes of wheat and within the three sub-genomes. Genes in highlighted boxes represent tandem duplications. (**A**) Physical map showing the chromosomal distribution, with position on the left side and the name of the gene on right side. (**B**) Distribution of HSP genes across 21 chromosomes. (**C**) distribution of HSP genes in the three sub-genomes.

A total of 753 HSP genes were identified in the wheat genome which includes 169 *TaSHSP*, 273 *TaHSP40*, 95 *TaHSP60*, 114 *TaHSP70*, 18 *TaHSP90* and 84 *TaHSP100*. This number is quite large compared to the HSPs reported previously in other plants such as *Arabidopsis*, foxtail millet and rice (Table 1). This might be due to the large genome size and higher ploidy level of wheat. Modern wheat is composed of three closely related and independently maintained genomes acquired from hybridization events. These three genomes are referred to as A, B and D sub-genome depending on their origin. The *TaHSP* genes are present on all the 21 wheat chromosomes (Fig. 1A, Table S1). A total of 17 *TaHSP* genes were not mapped as they could not be assigned to any chromosome. *TaHSP* genes showed a slightly higher presence on B and D sub-genomes (Fig. 1B,C). Maximum *TaHSP* genes i.e. 255 were mapped on the chromosomes of D sub-genome. Homoeologous chromosomes of group 7 had the highest density of *TaHSP* genes where chromosome 7D had 57 and 7 A & 7B had 48 *TaHSP* genes each. Chromosome 2A had the least number of *TaHSP* genes i.e 19. In the *TaSHSP* gene sub-family, chromosomes 3D and 7D had the maximum and 2A had the minimum density. *TaHSP*60 genes showed almost equal distribution among the 3 sub-genomes. On the other hand, *TaHSP*90 genes were absent on chromosomes 1, 3, 4, and 6. Overall, no sub-genome level specificity was observed in the distribution of HSP family. Similar observations have been reported in rice^16^ and foxtail millet^15^.

Gene duplication has been recognized as one of the major factor for gene family expansion. A duplicated gene can be retained as it is and perform the same function as identical copy or it can evolve into a gene with novel function. To understand it in the context of polyploid plants with large genomes, we looked at tandem duplication events in the *TaHSP* family. We found 65 tandem duplication events, where maximum tandem duplications were on chromosome 7D. There was no tandem duplication on chromosomes 2B and 2D, all other chromosomes had at least one tandemly duplicated gene pair. These duplications have been highlighted on the chromosomes in Fig. 1. Thirty tandem duplication events were identified in *TaSHSP*s which accounts for almost 45% of the identified *TaSHSP*s. Similarly, 25% of *TaHSP70s* and *TaHSP*100s were from tandem duplication events. There were no tandem duplications in *TaHSP90* family and only one duplication in *TaHSP*60 family. This suggests a strong selection pressure on the *TaHSP90* and *TaHSP60* genes which maintains the gene family size and probably its expression. *TaHSP40s* although being the largest HSP sub-family in wheat had only 11 tandem duplication events accounting for over 12% of the identified *TaHSP40s*. However, the tandemly duplicated arrays were largest in case of *TaHSP40* sub-family followed by *TaSHSP* family. Most tandem duplication identified in *Arabidopsis* or rice contains only two genes. Duplicated cluster containing more than three genes are rare in nature^19^. In case of *TaHSP*40s, these clusters had up to seven genes; *TaSHSP*s had up to five genes in one cluster. Other *TaHSP* families had arrays having two-three genes. Number of tandem duplication on individual chromosomes ranged from 1–4. Chromosome 4B, 7 A and 7D had 5 such events whereas 7D had 10 tandem duplication events, which was maximum among all the chromosomes. Chromosome 7 A, 7B and 7D also contained the largest duplicated arrays having 5–7 genes which was either of *TaSHSP* or *TaHSP40* genes. Tandem duplication seems to be crucial for the expansion of *TaHSP* family. Many of these duplicated genes can take on different expression profiles. We also found identical duplicates with a 100% sequence identity. We found 7, 8, 5, 7, 1, 5 identical duplicates in TaSHSP, TaHSP40, TaHSP60, TaHSP70, TaHSP90 and TaHSP100 sub-families, respectively. These duplicate pairs are given in Table S4.

The *TaSHSP* gene sub-family was identified by the presence of α-crystallin domain (pfam00011) and all *TaSHSPs* had at least one α-crystallin domain (Table S2) which acts as the signature motif. Proteins possessing the α-crystallin domain bind non-native proteins and are among the first to respond during stress conditions^20^. TaHSP60, TaHSP70 and TaHSP90 proteins were identified by the presence of characteristic domains *viz*., pfam00118 (Cpn60_TCP1), pfam00012 (HSP70) and pfam00183 (HSP90), respectively (Supplementary Fig. S1). All members of TaHSP90 family had another domain called HATPase_c (pfam02518), except TaHSP90.8.1, TaHSP90.11.1 and TaHSP90.12.2. This domain is found in many proteins possessing ATPase activity. TaHSP100 family members showed presence of at least five characteristic domains in all the members. These domains include Clp_N (pfam02861), ClpB_D2-small (pfam10431) and variants of AAA domain (pfam07724 and pfam07728) (Table S2). These domains perform the cumulative function of re-solubilizing denatured protein aggregates using ATP hydrolysis^21^. Some TaHSP100 proteins such as TaHSP100.6.1, TaHSP100.6.2, TaHSP100.9, TaHSP100.11, and TaHSP100.20 had presence of UVR domain (Table S2). This domain is present in proteins involved in DNA repair mechanisms and has also been reported in HSP100s of rice and foxtail millet^15,22^. TaHSP70s and TaHSP60s also showed presence of MreB_Mbl and PIP5K domains, respectively which have also been reported in foxtail millet^15^. The presence of non-HSP domains in some TaHSPs suggests an alternative or modified function, apart from or during protein modification.

### Gene structure organization

Structure of a gene determines its coding potential and can also give hints about the ancestry of genes since genes with similar structure probably evolved from a common ancestor. Diverse variation in gene structure organization in terms of gene length, number of introns and number of exons was observed in different *TaHSP* sub-families (Supplementary Fig. S2). *TaSHSP* sub-family differed greatly in terms of gene structure from rest of the *TaHSP* sub-families as most of them were intronless. Maximum two introns were found in the *TaSHSP* sub-family. Introns are vital component of gene which are known to perform significant roles in gene expression regulation and stability through intron mediated enhancers, alternative splicing and by increasing the natural selection efficiency^23,24^.

We had observed some intronless genes in the different *TaHSP* sub-families, but none of the *TaHSP60* and *TaHSP90* sub-family genes were intronless. Usually, newly evolved genes or pseudogenes arising from retro-transposition^25^ are intronless, which arise by copy-paste type mechanism^24^. *TaSHSP* sub-family had the maximum number of intronless genes followed by *TaHSP40* sub-family. *TaHSP90.7, TaHSP90.10* and *TaHSP90.4* had 19 introns, which was the highest among all *TaHSP* sub-families. *TaHSP40.257* and *TaHSP60.91* had the smallest and largest gene size of 280 bp and 31800 bp, respectively while their protein lengths were 109 and 1801 amino acids, respectively. Largest protein was from the *TaHSP40* sub-family; TaHSP40.33 which had 2577 amino acids whereas TaHSP100.50.1 was the smallest protein, having just 70 residues. *TaHSP* genes were almost equally distributed on both the positive and negative strands. Some of the *TaHSP* genes identified in this study could be pseudogenes^26^ however; to ascertain which *TaHSP*s are pseudogenes, experimental evidences are required. As the characteristic feature of pseudogenes is that they do not express^27^, we took TPM values of all the *TaHSP*s across all the 36 transcriptome experiments available at expVIP database. The expVIP database contains transcriptome experiments conducted on different kinds of abiotic and biotic stresses, tissue specific expression and different developmental stages. *TaHSPs* whose sum of TPM across all these experiments was less than one were considered as potential pseudogenes. Interestingly, 119 out of 169 *TaSHSP*s satisfied these criteria. List of these potential pseudogenes is given in Table S4. All other *TaHSP*s showed significant expression in at least one of the available studies except 2 *TaHSP40*s; *TaHSP40.125* and *TaHSP40.186*. Most of these *TaSHSP*s and the two *TaHSP40*s are intronless and are also part of tandemly duplicated arrays. This suggests that pseudogenization and tandem duplication had played a major role in the expansion of *TaSHSP* gene sub-family most of whose members are potential pseudogenes scattered throughout the genome. Number of potential *TaSHSP* pseudogenes was similar across the three sub-genomes.

### Gene Ontology (GO)

Gene ontology helps in the functional analysis of genes by determining its similarity with other genes of known function. It improves the annotation of genes. All *TaHSP*s were successfully annotated and assigned GO terms (Table S3). BLAST2GO top blast-hit distribution depicts maximum similarity of *TaHSP*s with *Aegilops tauschii* (Supplementary Fig. S3). *TaHSP*s were also annotated again using AgriGO^28^ for further validation (Supplementary Figs. S5, S6) which provided similar results as BLAST2GO. Among the biological process category, all TaHSP sub-families enriched for response to protein folding (GO: 0006457) and various stress response categories (Supplementary Figs. S4 & S5). In the cellular component category, all TaHSP families showed enrichment by multiple organelles of the cell (Supplementary Figs. S4 & S6). Sub-cellular localization prediction carried out by using BUSCO (Supplementary Fig. S7) also gave similar results.

In the molecular function category, protein binding (GO: 0005515) was the most enriched category followed by ATP binding (GO: 0005524) (Supplementary Figs. S4 & S8). ATP is bound by the chaperone in association with a metal ion(s)^29^. Apart from protein folding and stress response, the GO term enrichment suggested multiple roles of TaHSPs in the cell such as transport of protein, growth and development related processes, immune responses, metabolism, etc.

### Cis-acting regulatory elements (CAREs)

**Figure 2:**
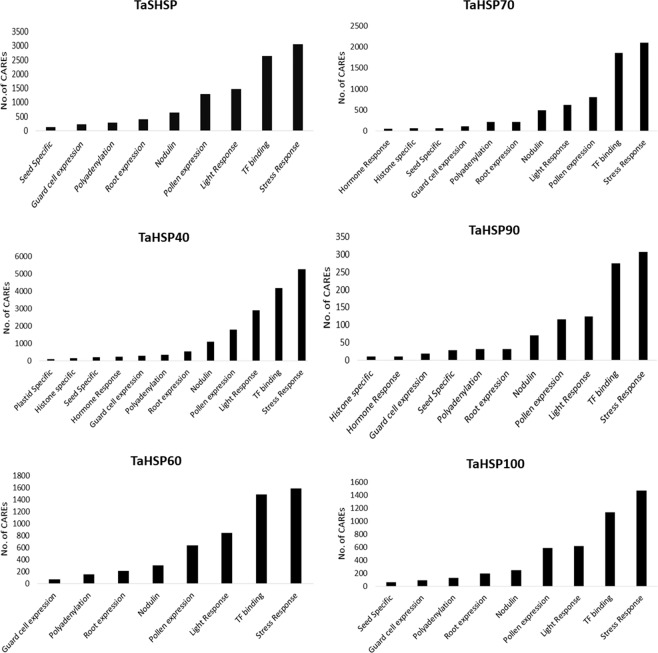
Most commonly occurring *cis-*acting regulatory elements in different *TaHSP* sub-families.

CAREs are non-coding DNA elements present in the promoter region of genes. These are involved in the regulation of gene expression and are important components of gene regulatory networks. They also give an idea about the physiological process, that which gene might be involved. A total of 212 unique CAREs were identified in the *TaHSP* gene family (Table S5) out of which 186, 184, 163, 163, 123 and 158 motifs were predicted in TaSHSP, TaSHSP40, TaHSP60, TaHSP70, TaHSP90 and TaHSP100 sub-families, respectively. In any TaHSP sub-family, CAREs involved in stress responses and transcription factor (TF) binding were the most abundant ones (Fig. 2). Stress responses include both biotic and abiotic stress responses. *TaHSP*s had motifs to bind different TFs such as MYB, MYC, bZIPs, etc. This suggests that *TaHSP*s play a crucial role in stress responses and these responses are regulated by multiple TFs. TFs involved in developmental processes and hormone responses also had binding sites in *TaHSP* promoters. Light responsive CAREs were also prevalent in all TaHSP sub-families. These are those CAREs that are involved in photosynthesis/non-photosynthesis mediated light responses as well as circadian rhythm based light responses. *TaHSP*s also had CAREs responsible for tissue specific expression. Guard cell specific CAREs were present in all *TaHSP* sub-families. CAREs which induces expression specifically in root and seed were also present in all sub-families. Polyadenylation signals were also prevalent in all sub-families; these signals are often involved in alternative polyadenylation which leads to the production of transcripts with differing 3′-UTR length. This, in turn, leads to differing regulatory and protein-coding potential of those transcripts^30^ The CAREs present in *TaHSP*s suggest that *TaHSP*s are not just stress responsive but are also involved in many other processes that can be regulated by light, hormone or developmental stages. Although it can be said that stress response is among the primary functions of *TaHSP*s.

### **Expression profiling of*TaHSP*genes during different developmental stages and stress conditions**

**Figure 3:**
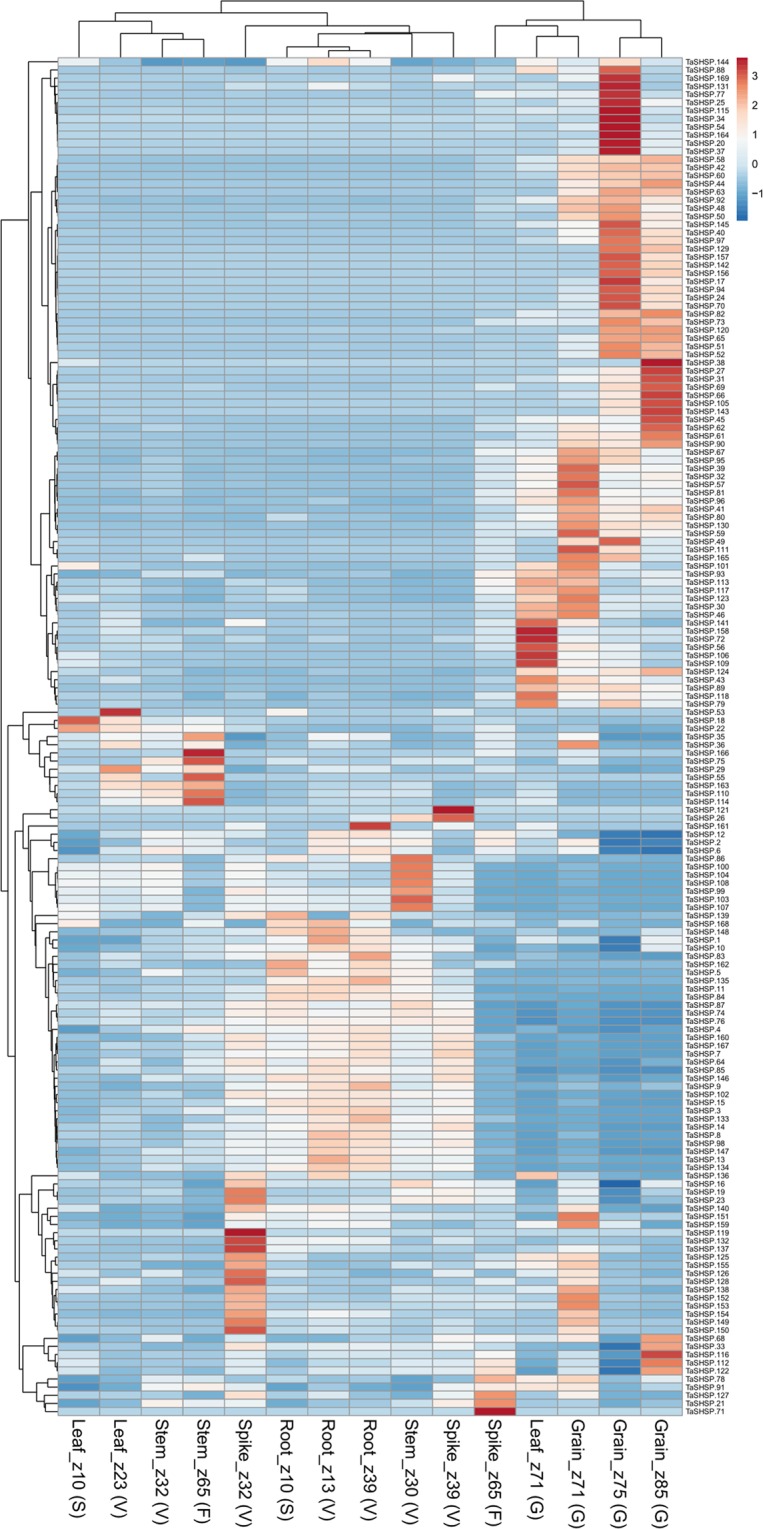
Heatmap representing the expression of all the *TaSHSP* genes in various developmental stages. TPM values were centered and scaled by unit variance scaling method to create the heatmap.

**Figure 4:**
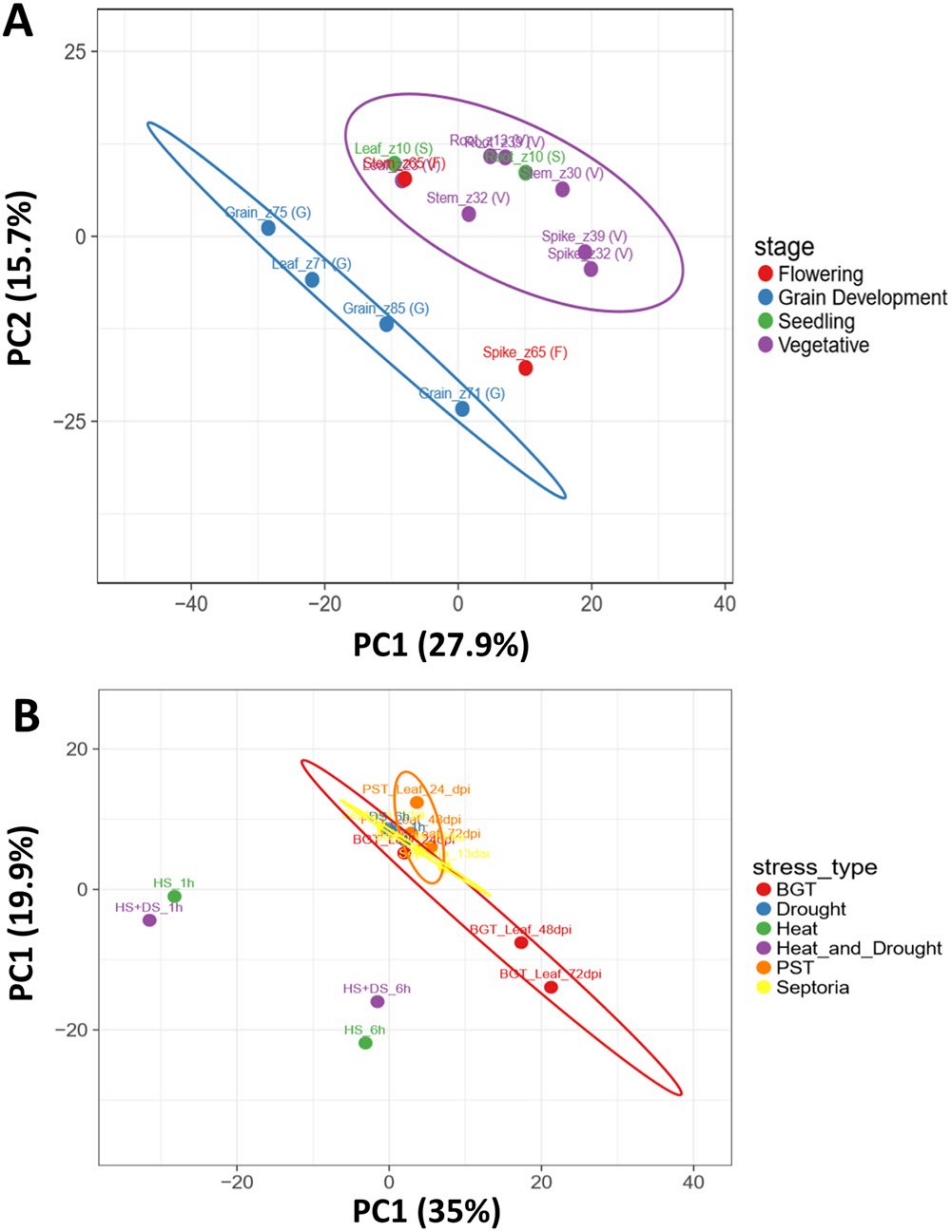
PCA plots showing grouping of various (**A**) Development stages (**B**) Biotic and abiotic stress on the basis of HSP expression pattern.

To understand how *TaHSP*s are involved in development or stress responses, we retrieved TPM values of all *TaHSP*s from experiments involving abiotic/biotic stress as well as different developmental stages from the expVIP database. These TPM values were used to perform Principal Component Analysis (PCA) and create heatmaps (Supplementary Fig. S9). Five different tissues at three different developmental time points were taken for this study. The time points are the Zadoks scale. Different *TaHSP* sub-families show differential induction in different tissues. *TaHSP*s such as *TaHSP100.22*, *TaHSP100.63*, *TaHSP100.72*, *TaHSP70.21*, *TaHSP70.51*, *TaHSP70.58* etc. showed induction at spike z39 stage. *TaHSP*40.230, *TaHSP*40.232, *TaHSP*40.243 etc. were up regulated in leaves at z71 stage. Multiple *TaHSP70s* were induced at different spike stages especially z39 stage. *TaHSP70.19*, *TaHSP70.22* and *TaHSP70.25* were induced at root z10 stage. This suggests different *TaHSPs* might be involved in development of different tissues at different stages. Many *TaSHSPs* showed induction at the three stages of grain tissue, most were induced at z75 stage compared to the other two. *TaSHSP17*, *TaSHSP20*, *TaSHSP34*, *TaSHSP37* etc. were highly induced at grain z75 stage. The decrease in *TaSHSP* expression at grain z85 stage suggested that as the grain matures *TaSHSPs* get down regulated (Fig. 3). Accumulation of *TaSHSPs* in developing seed has been reported earlier^12,13^. Protective role of HSPs during seed development has also been reported earlier^31^. The PCA plot of different developmental stage expression profiles also suggests that *TaHSPs* are involved in grain development. Subsequently, grain tissue expression profiles cluster separate from other expression profiles (Fig. 4A and Supplementary Fig. S9). Very few members of the *TaHSP60* and *TaHSP90* sub-family showed induction during any of the different developmental stages. *TaHSP60.2* was induced at grain z71 stage, *TaHSP60.86*, *TaHSP60.95* among others were induced at leaf z10 stage. *TaHSP90.5* and *TaHSP90.11* were induced at leaf z10 stages.

**Figure 5:**
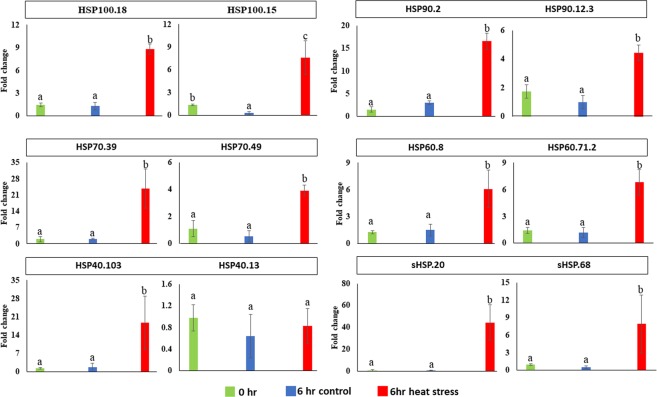
Quantitative real time PCR based expression validation of selected TaHSP genes. Single letters over bars represent results of Tukey HSD test. Means not sharing a letter are significantly different (p-value <0.05).

Expression profiling of *TaHSPs* was also performed for three different stress conditions i.e. biotic stress, heat stress, and drought stress. Heat shock proteins are historically known to be induced during heat stress (Supplementary Fig. S10). Several members of the *TaSHSP*, *TaHSP100*, *TaHSP70* and *TaHSP90* sub-families were found to be induced during abiotic stress, however, *TaHSP40s* and *TaHSP60s* were induced more under biotic stress. Apart from a few exceptions, these two sub-families instead showed down regulation upon abiotic stress. There were some *TaHSPs* that were highly induced during infection by any of the three pathogens, for example, *TaSHSP127* is highly induced upon *Septoria* infection. *TaHSP70.53* and *TaHSP90.14* show high induction during *Blumeria* infection. In the case of abiotic stress, the data suggests that HSP-mediated response is more prevalent during the initial hours of heat stress. All *TaHSP*s respond similarly to ‘heat stress’ or ‘heat and drought combined’ stress. It seems that *TaHSPs* are not involved in drought stress, which is the reason that expression profiles of ‘heat stress’ or ‘heat and drought combined’ are similar. This is also evident from the PCA plots (Fig. 4B and Supplementary Fig. S9) where HS and HS + DS expression profile cluster together. Expression patterns of some selected *TaHSPs* were also validated by qRT-PCR at seedling stage heat treatment which were in line with the RNAseq results (Fig. 5). Overall the data revealed a primary role for many *TaHSPs* in different kinds of stress responses.

Interestingly, many of these *TaHSPs* are tandem duplicates or are homeologues. Most of them show similar expression with only slight variation. However, some homeologues show extremely different expression patterns, for example, *TaSHSP.68*, *TaSHSP.80* and *TaSHSP.90* are homeologues but unlike *TaSHSP80* and *TaSHSP92*, *TaSHSP68* shows high expression levels at ‘heat stress 1 h’ time point. Similarly, *TaHSP70.51*, *TaHSP70.59* and *TaHSP70.66* are also homeologues but unlike the other two, *TaHSP70.51* shows very high expression levels at spike z39 stage. This suggests that some homeologues might have evolved to take on new physiological roles.

### Physico-chemical properties

HSP family is divided into sub-families based on of their molecular weight (MW). However, not all *TaHSPs* of any particular family have identical molecular weights (Table S6). *TaHSP40*, *TaHSP60*, *TaHSP70*, *TaHSP90*, *TaHSP100* and *TaSHSP* sub-families had average molecular weight of 49.97, 101.09, 67.49, 80.26, 81.62 and 23.90 kDa, respectively. The average isoelectric point (pI) of *TaHSP* sub-families was between 4.9 and 7.86; *TaHSP40* had the highest average pI, 7.86 while *TaHSP90* sub-family had the lowest average pI of 4.9. Two-dimensional electrophoresis based proteomic analysis of TaHSP proteins or heat shock response in wheat^32^ and barley^33^ have yielded HSPs in similar pI range. We plotted MW of TaHSPs with their pI to view the MW distribution of different TaHSP sub-families (Supplementary Fig. S11). The plots suggest that although not all TaHSPs from a particular family have similar MW, most TaHSPs clusters together because they have similar molecular weights. Average isoelectric point (pI) of all sub-families ranged from 4 to 12 (Supplementary Fig. S11). Minimum and maximum pI, 4.304 and 12.20 were of TaSHSP23.1 and TaHSP40.248 respectively (Table S6).

### **Phylogeny and homologs of*TaHSPs*in related species**

**Figure 6:**
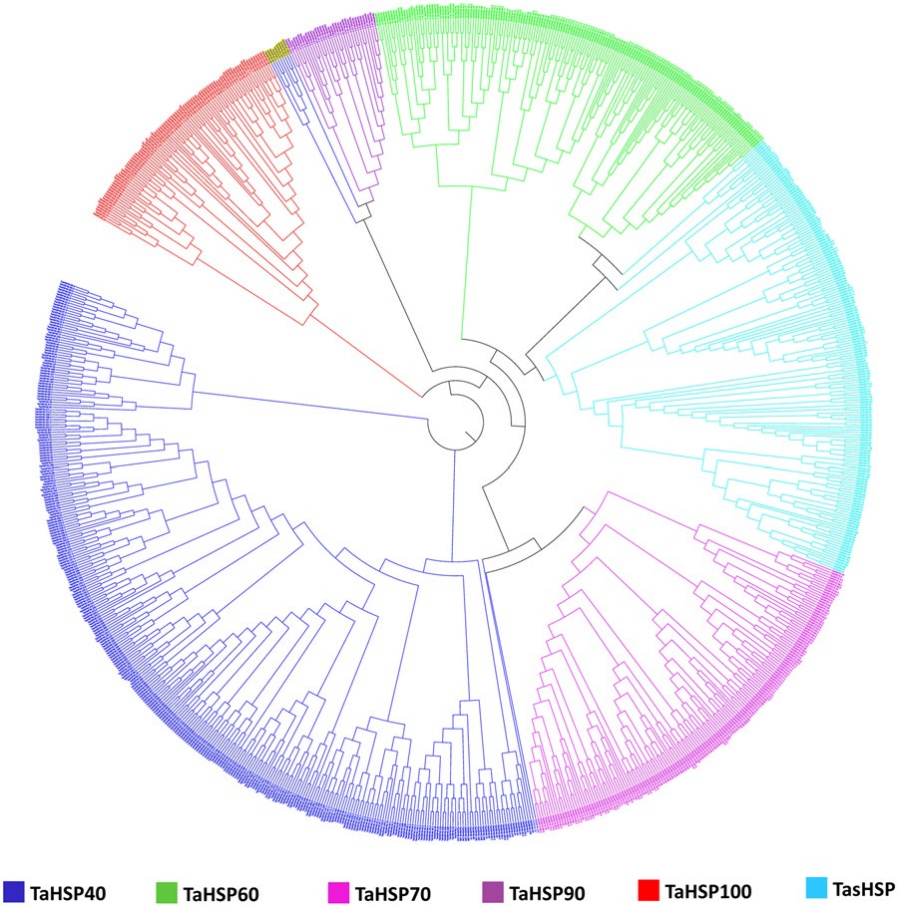
Phylogenetic relationship within HSP sub-families in wheat, inferred using maximum likelihood method. Different colors represent different sub-families within the HSP family.

Phylogeny helps in understanding how genes diverge in the course of evolution. Phylogenetic relationship was inferred between 753 identified TaHSP proteins. All TaHSP proteins were grouped within their respective subfamilies except some members of the TaHSP40 sub-family (Fig. 6, Supplementary Fig. S12). The *TaHSP40s* which group with *TaHSP100s* are either intronless or have very large introns and very short exons. They could be pseudogenes and this could be the reason why they did not group together with other *TaHSP40s* due to a lack of overall sequence similarity. Tandem duplicated genes often cluster together in a single clade^34^. This is also true in case of *TaHSPs*.

**Figure 7:**
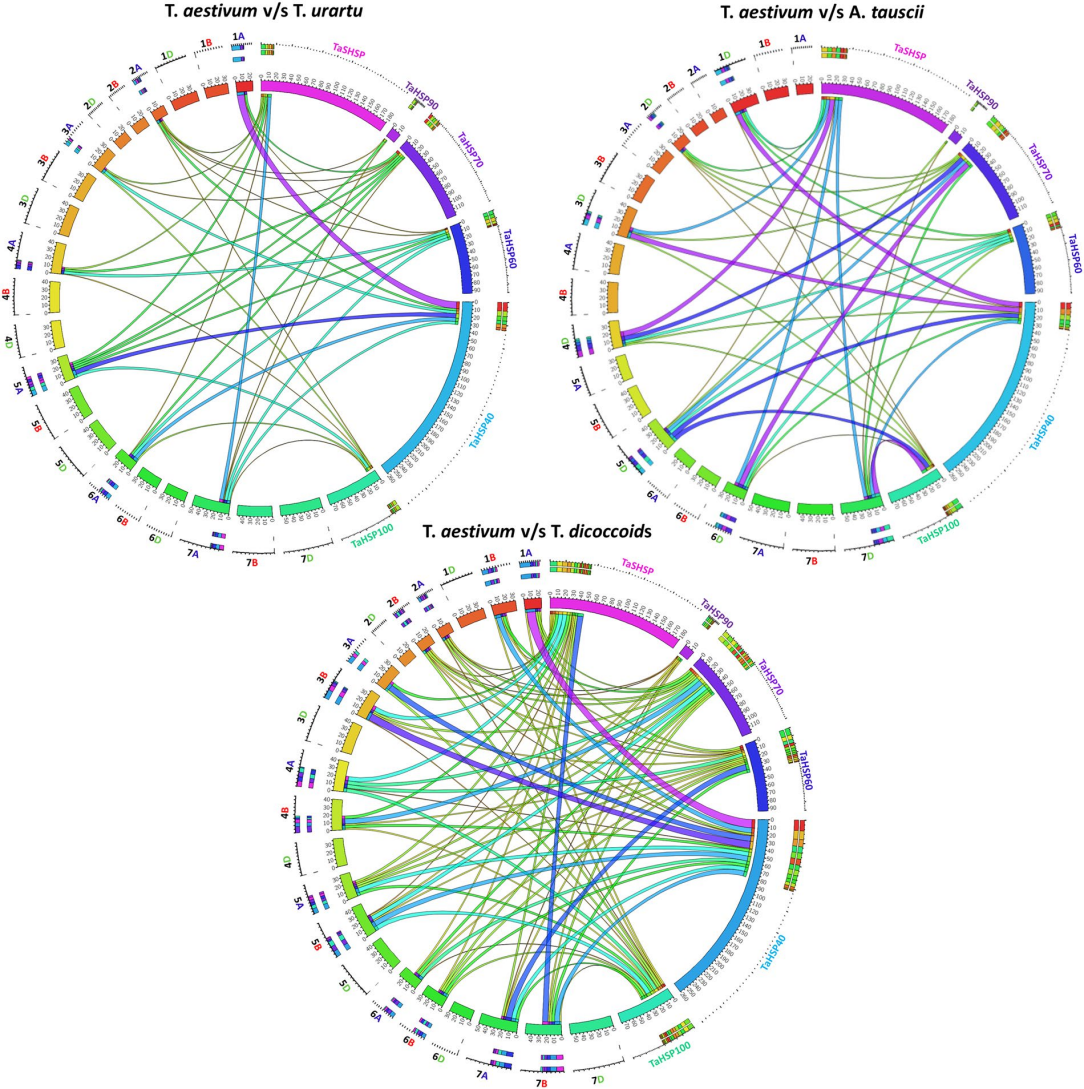
Syntenic relationship of *TaHSP*s orthologous with its close relative’s *viz., Ae. tauschii, T. urartu* and *T. dicoccoides*.

Since wheat evolved by hybridisation, we further tried to understand the syntenic relationship between wheat and its genome donors. Orthologous *TaHSP* genes were sorted chromosome-wise in *T. urartu*, *Ae. tauschii* and *T. dicoccoides* and were represented by Circos circular ideogram (Fig. 7). As expected, a high level of conservation was observed in *HSP* genes of *Ae. tauschii*, *T. urartu* (D- and A-genome donors of wheat) and *T. dicoccoides* (AB genome). It was observed that up to 20–60% of *TaHSP* genes of each chromosome were syntenic with their respective chromosomes on *T. urartu* (A homoeologs) and *Ae. tauschii* (D homoeologs) but, this percentage was higher in *T. dicoccoides* (a tetraploid) (Fig. 7). This shows that some of the wheat HSP genes have synteny with their progenitors but in course of evolution and with polyploidization, their number increased by gene complementation to confer adaptive plasticity^35^. Orthologous gene search in *O. sativa, Hordeum vulgure, Sorghum bicolor, Zea mays* and *A. thaliana* also indicated conservation in HSPs among unrelated monocots and dicots (Supplementary Fig. S13). Interestingly*, A. thaliana* showed orthologous relationship with every subfamily of *TaHSP* whereas *H. vulgure* showed orthologous relationship with only three sub-families (*TaSHSP*, *TaHSP90* and *TaHSP70*) (Supplementary Fig. S13). Maximum numbers of orthologs were observed in *Z. mays* (with average percent identity of 91.6%) followed by *O. sativa* (92.4%), *S. bicolor* (92.4%)*, A. thaliana* (90.7%) and least orthologs were present in *H. vulgure* (95.9%) (Table S7). It has also been observed that sub-family *TaHSP70* showed the highest number of orthologs and maximum synteny with others, while *SHSPs* sub-family showed least orthology and minimum synteny. Overall, *TaHSP70* was found to be highly conserved and *TaSHSP* least conserved subfamily in course of plant evolution.

## Conclusion

With the increasing concerns about global warming and rising earth temperatures, it is very important to know about proteins that provide thermo-tolerance in crop plants. HSP family is one such gene family that is involved in tolerance against heat stress. The present study identified and characterized the HSP family in wheat genome, all six HSP sub-families were identified. Tandemly duplicated genes and potential pseudogenes were also identified. Expression profiling revealed the role of *TaHSPs* during different developmental stages and stress responses. *TaHSP40s* and *TaHSP60s* were induced more in response to biotic, whereas other *TaHSP* sub-families were induced more in response to abiotic stress. *TaSHSP* were also prevalent during grain development. The study provides candidate genes for improving development stage-specific thermo-tolerance and also enables better understanding of the seed development process in bread wheat.

## Materials and Methods

### Identification and distribution of heat shock proteins in wheat genome

Two approaches were used to identify heat shock proteins in wheat, domain based and homology based. In the first approach PFAM identifiers of domains present in HSPs (Table S1) were taken from previous literature and their Position Specific Scoring Matrices (PSSMs) were downloaded from NCBI Conserved Domain Database (NCBI CDD) (ftp://ftp.ncbi.nih.gov/pub/mmdb/cdd/cdd.tar.gz)^36^. These PSSMs were used to create a database for Reversed Position Specific BLAST (RPS-BLAST). *T. aestivum* proteome was downloaded from Ensembl Plants (http://plants.ensembl.org/index.html) and used as a query in RPS-BLAST against the locally made database.

In the second approach^37^, the protein sequences of HSPs known in related plant species were downloaded from the HSPIR database (http://pdslab.biochem.iisc.ernet.in/hspir/index.php) and BLASTP search was performed against the bread wheat protein sequences with an e-value cut-off of 0.0001 and bit-score>100. Based on both the above approaches, potential HSP candidates were identified.

After removing redundant results the final sequences were confirmed for the presence of HSP related domains using other databases- HMMscan (https://www.ebi.ac.uk/Tools/hmmer/search/hmmscan), InterPro (https://www.ebi.ac.uk/interpro) and NCBI CDD (https://www.ncbi.nlm.nih.gov/Structure/cdd/cdd.shtml). For localization on chromosomes, genomic coordinates of HSP genes were retrieved from Ensembl plants biomart (http://plants.ensembl.org/biomart/martview). The genes were named with a prefix ‘Ta’ for HSP40, HSP60, HSP70, HSP90, HSP100 and ‘TaS’ for small HSP, and numbered in increasing order with their increasing position on chromosome going from the short arm to long arm. The nomenclature of HSP genes was in agreement with the available guidelines (https://wheat.pw.usda.gov/ggpages/wgc/98/Intro.htm). MapChart v2.3^38^ was used to represent various *TaHSP* genes on the 21 wheat chromosomes.

Genes that were presenting adjacent to each other were screened for potential tandem duplication events. Gene pairs that were present on the same strand and whose amino acid identity was more than 40% and were intervened by less than five genes were considered as tandem duplication events.^39^

### **Gene structure, gene ontology, and*cis-*acting regulatory elements (CAREs) identification**

Intron, exon positions, and gene structure were represented and analyzed using GSDraw (https://wheat.pw.usda.gov/piece/GSDraw.php). For gene ontology, *TaHSP* protein sequences were BLAST searched against the NCBI NR database and the results were used to predict gene ontology terms using BLAST2GO^40^, EggNogg (http://eggnogdb.embl.de/#/app/emapper) and agriGO^41^. For identification of CAREs, sequences 1500 bp upstream of HSP genes were retrieved from Ensembl plants and analyzed using PLACE database (https://www.dna.affrc.go.jp/PLACE/?action=newplace). Number of occurrences for each CARE motif was counted for each *TaHSP* sub-family and the most frequently occurring motifs were used to make Fig. 2. Conserved motifs for each *TaHSP* sub-family were identified using Meme tool (http://meme-suite.org/tools/meme) with default settings except the motif dimensions which were kept at, minimum width 5 and maximum width 50 amino acid^42^.

### RNA-seq derived gene expression profiling

Transcripts per million (TPM) value were taken for each *TaHSP* from expVIP database (http://www.wheat-expression.com/). Heatmaps and PCA plots were generated using clustvis (https://biit.cs.ut.ee/clustvis/)^43^.

### Plant material, growth conditions and heat treatment

Six day old wheat seedlings were acclimatized for two days at growth chamber conditions with artificial light system. They were further subjected to heat treatments (37 °C) for 6 hr. Controls were kept at ambient conditions (24 °C). After treatment seedlings were harvested for RNA isolation and kept at −80 °C until downstream experiments.

### RNA isolation and Real-Time PCR

Seed tissue from control and heat-treated plants were used for isolation of total RNA using the TRIZOL^®^ reagent (Ambion, USA) as described by Chomczynski^40^. Real-time PCR reactions were carried out using the ABI 7500 Fast Real-Time PCR (Applied Biosystems). Each reaction was carried out in triplicates with three biological and technical replicates. Amplification was performed according to the default cycle and fold change values based on average 2^-ΔΔ^CT values^41^ were used for plotting graphs.

### Physico-chemical properties and subcellular localization

Isoelectric point and molecular weight of TaHSP proteins were analyzed using isoelectric point calculator (isoelectric.org). Subcellular localization was predicted using BUSCA^44^.

**Phylogenetic analysis and homologous gene search**. Protein sequences were used to perform multiple sequence alignment using CLUSTALW and phylogenetic trees were prepared using RAxML^45^ with 1000 bootstrap. For identification of orthologous genes in close relatives of *T. aestivum*, CDS sequences of *T. urartu* (ftp://ftp.ensemblgenomes.org/pub/plants/release-42/fasta/triticum_urartu), *Ae. tauschii* (ftp://ftp.ensemblgenomes.org/pub/release-42/plants/fasta/aegilops_tauschii/cds) and *T. dicoccoides* (ftp://ftp.ensemblgenomes.org/pub/release-42/plants/fasta/triticum_dicoccoides/cds/) constituting A, D and AB genome, respectively were retrieved from Ensembl plants and subjected to BLASTN search against *TaHSP* CDS sequences. The results were filtered using an e-value cutoff of 1E-10 and 150 bit-score cut-off. The top hits were considered as orthologs in that species. Similar approach was used for orthologous gene search in other monocots and dicots *viz., O. sativa, H. vulgure, S. bicolor, Z. mays* and *A. thaliana*. Synteny relationship of HSP gene family within and among different species was performed using Circos online server (http://mkweb.bcgsc.ca/tableviewer/visualize/).

## Supplementary information

Supplementary Figures.

Dataset 1.

Dataset 2.

Dataset 3.

Dataset 4.

Dataset 5.

Dataset 6.

Dataset 7.
