# Supplementary material for: Genome-wide Identification and Characterization of Heat Shock Protein Family Reveals Role in Development and Stress Conditions in Triticum aestivum L
Source: Sci Rep. 2020 May 12;10:7858. doi: 10.1038/s41598-020-64746-2 (PMC7217896; doi:10.1038/s41598-020-64746-2)
Supplement: Supplementary file 1 — Supplementary Figures. [file 41598_2020_64746_MOESM1_ESM.pdf]

Genome-wide Identification and Characterization of Heat Shock Protein Family Reveals Role in Development and Stress Conditions in *Triticum aestivum* L.

Ashish Kumar[1†], Saloni Sharma[2†], Venkatesh Chunduri[2], Amandeep Kaur[2], Satinder Kaur[3], Nikhil Malhotra[2], Aman Kumar[2], Payal Kapoor[2], Anita Kumari[2], Jaspreet Kaur[4], Humira Sonah[2]* and Monika Garg[2]*

[1] South Asian University, Chankyapuri, New Delhi, India 110021

[2]Agri-Biotechnology Division, National Agri-Food Biotechnology Institute (NABI), S.A.S. Nagar (Mohali), Punjab, India

[3]Punjab Agricultural University, Ludhiana 141004

[4]UIET, PU, Chandigarh-160014

[†]Contributed equally

***Corresponding author: biohuma@gmail.com and mkajgarg@gmail.com**

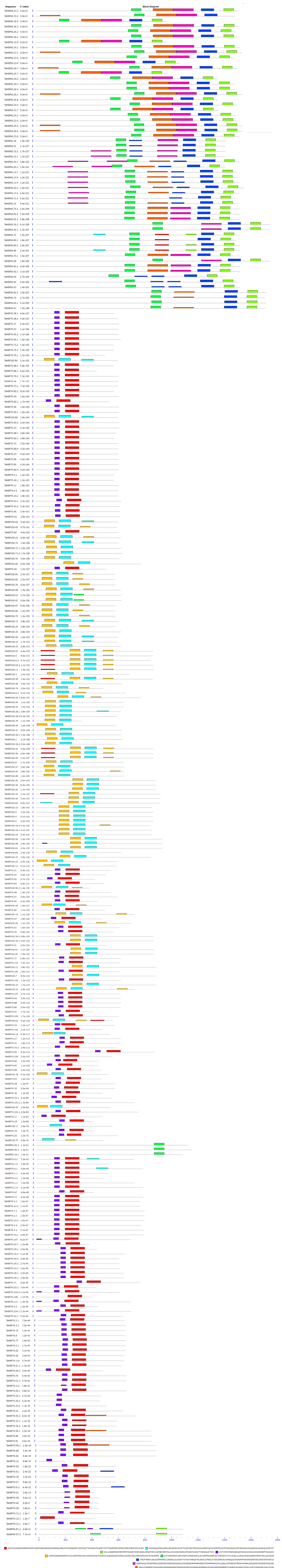

SFig. 1: Conserved Motifs of TaHSPs elucidated by MEME. Colored boxes representing different conserved motifs with different sequences and size.

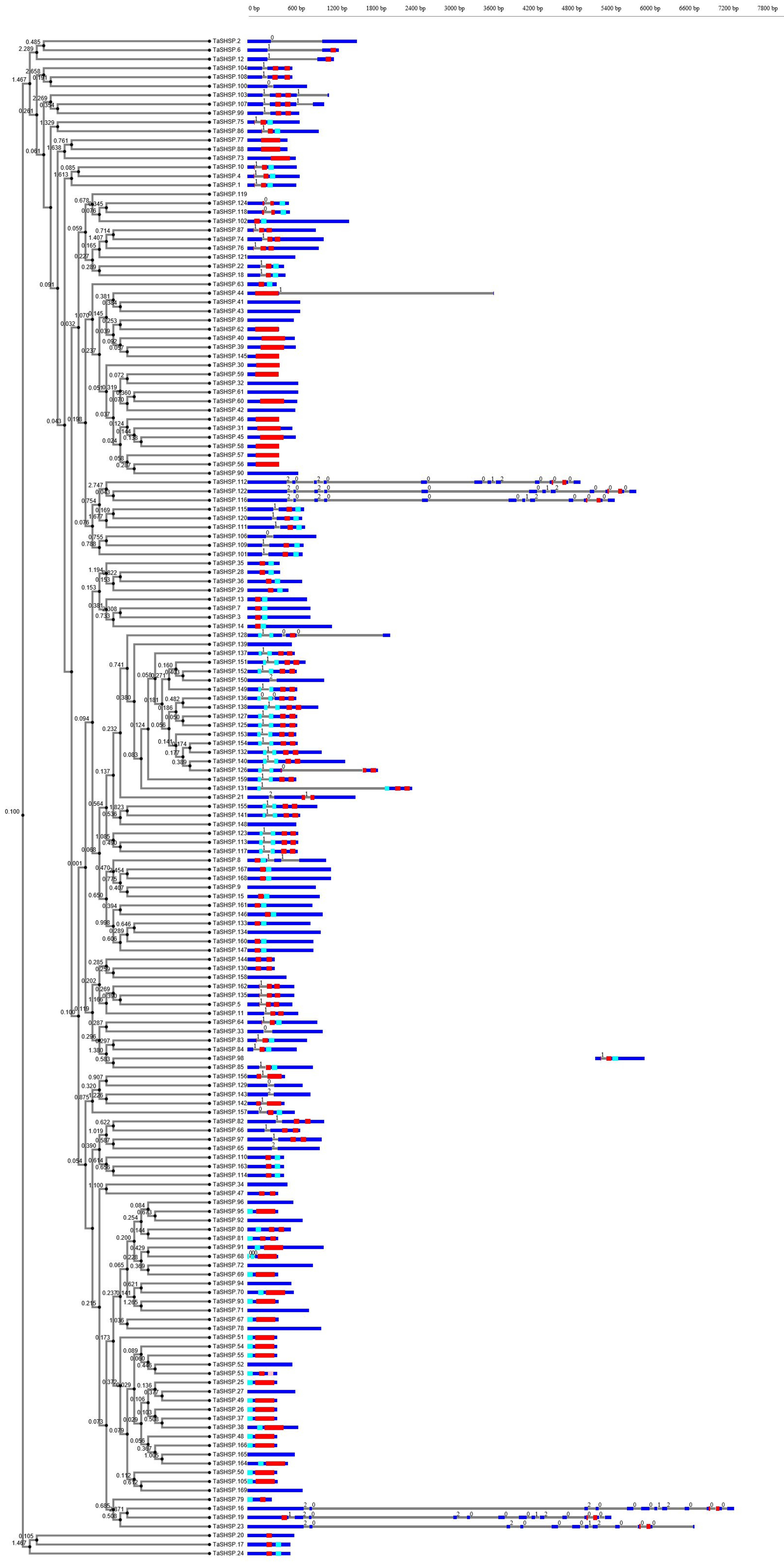

0 bp
600 bp
1200 bp
1800 bp
2400 bp
3000 bp
3600 bp
4200 bp
4800 bp
5400 bp
6000 bp
6600 bp
7200 bp
7800 bp
TaSHSP.2
TaSHSP.6
TaSHSP.12
TaSHSP.104
TaSHSP.108
TaSHSP.100
TaSHSP.103
TaSHSP.107
TaSHSP.99
TaSHSP.75
TaSHSP.86
TaSHSP.77
TaSHSP.88
TaSHSP.73
TaSHSP.10
TaSHSP.4
TaSHSP.1
TaSHSP.119
TaSHSP.124
TaSHSP.118
TaSHSP.102
TaSHSP.87
TaSHSP.74
TaSHSP.76
TaSHSP.121
TaSHSP.22
TaSHSP.18
TaSHSP.63
TaSHSP.44
TaSHSP.41
TaSHSP.43
TaSHSP.89
TaSHSP.62
TaSHSP.40
TaSHSP.39
TaSHSP.145
TaSHSP.30
TaSHSP.59
TaSHSP.32
TaSHSP.61
TaSHSP.60
TaSHSP.42
TaSHSP.46
TaSHSP.31
TaSHSP.45
TaSHSP.58
TaSHSP.57
TaSHSP.56
TaSHSP.90
TaSHSP.112
TaSHSP.122
TaSHSP.116
TaSHSP.115
TaSHSP.120
TaSHSP.111
TaSHSP.106
TaSHSP.109
TaSHSP.101
TaSHSP.35
TaSHSP.28
TaSHSP.36
TaSHSP.29
TaSHSP.13
TaSHSP.7
TaSHSP.3
TaSHSP.14
TaSHSP.128
TaSHSP.139
TaSHSP.137
TaSHSP.151
TaSHSP.152
TaSHSP.150
TaSHSP.149
TaSHSP.136
TaSHSP.138
TaSHSP.127
TaSHSP.125
TaSHSP.153
TaSHSP.154
TaSHSP.132
TaSHSP.140
TaSHSP.126
TaSHSP.159
TaSHSP.131
TaSHSP.21
TaSHSP.155
TaSHSP.141
TaSHSP.148
TaSHSP.123
TaSHSP.113
TaSHSP.117
TaSHSP.8
TaSHSP.167
TaSHSP.168
TaSHSP.9
TaSHSP.15
TaSHSP.161
TaSHSP.146
TaSHSP.133
TaSHSP.134
TaSHSP.160
TaSHSP.147
TaSHSP.144
TaSHSP.130
TaSHSP.158
TaSHSP.162
TaSHSP.135
TaSHSP.5
TaSHSP.11
TaSHSP.64
TaSHSP.33
TaSHSP.83
TaSHSP.84
TaSHSP.98
TaSHSP.85
TaSHSP.156
TaSHSP.129
TaSHSP.143
TaSHSP.142
TaSHSP.157
TaSHSP.82
TaSHSP.66
TaSHSP.97
TaSHSP.65
TaSHSP.110
TaSHSP.163
TaSHSP.114
TaSHSP.34
TaSHSP.47
TaSHSP.96
TaSHSP.95
TaSHSP.92
TaSHSP.80
TaSHSP.81
TaSHSP.91
TaSHSP.68
TaSHSP.72
TaSHSP.69
TaSHSP.94
TaSHSP.70
TaSHSP.93
TaSHSP.71
TaSHSP.67
TaSHSP.78
TaSHSP.51
TaSHSP.54
TaSHSP.55
TaSHSP.52
TaSHSP.53
TaSHSP.25
TaSHSP.27
TaSHSP.49
TaSHSP.26
TaSHSP.37
TaSHSP.38
TaSHSP.48
TaSHSP.166
TaSHSP.165
TaSHSP.164
TaSHSP.50
TaSHSP.105
TaSHSP.169
TaSHSP.79
TaSHSP.16
TaSHSP.19
TaSHSP.23
TaSHSP.20
TaSHSP.17
TaSHSP.24

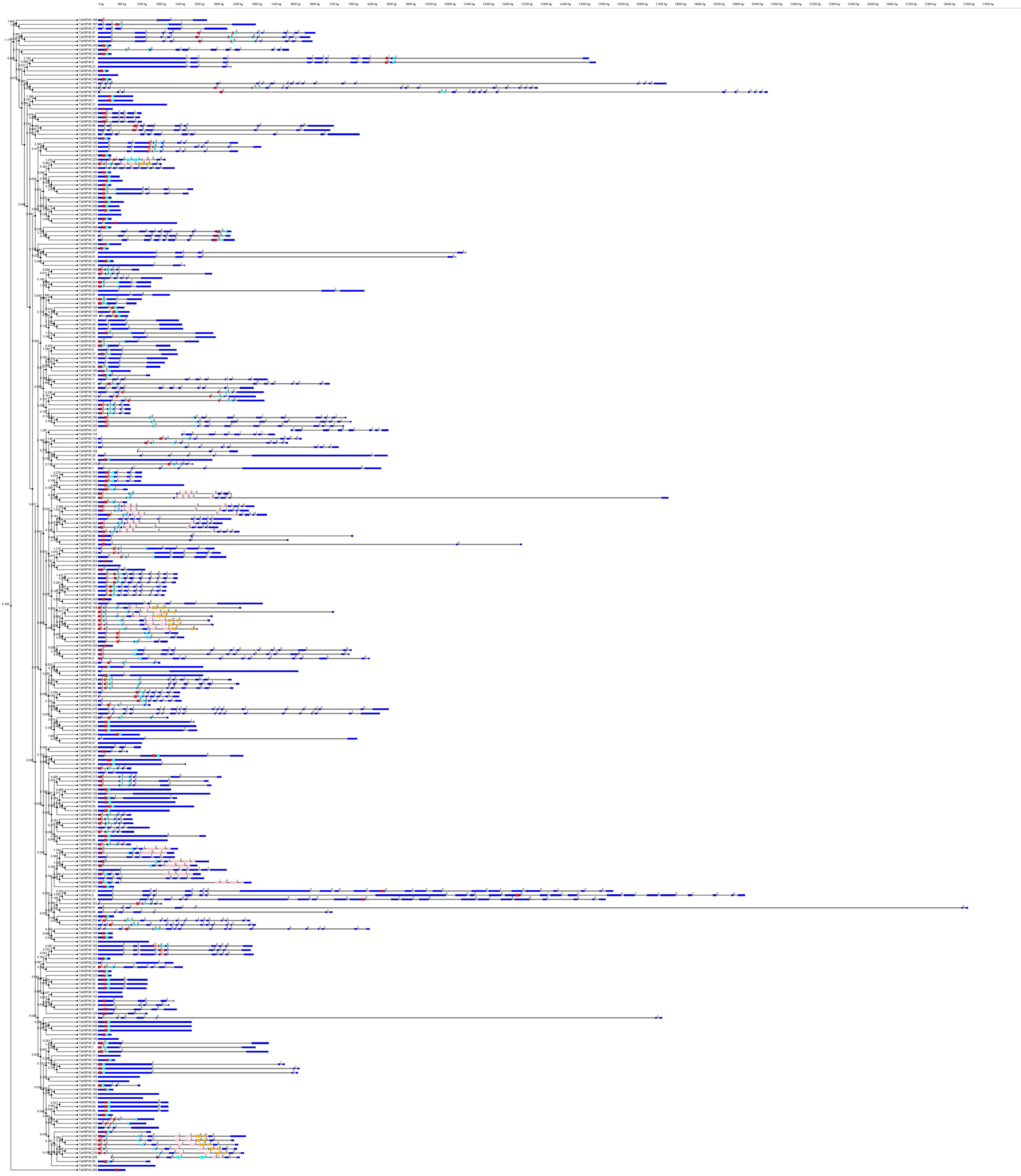
0 bp 600 bp 1200 bp 1800 bp 2400 bp 3000 bp 3600 bp 4200 bp 4800 bp 5400 bp 6000 bp 6600 bp 7200 bp 7800 bp 8400 bp 9000 bp 9600 bp 10200 bp 10800 bp 11400 bp 12000 bp 12600 bp 13200 bp 13800 bp 14400 bp 15000 bp 15600 bp 16200 bp 16800 bp 17400 bp 18000 bp 18600 bp 19200 bp 19800 bp 20400 bp 21000 bp 21600 bp 22200 bp 22800 bp 23400 bp 24000 bp 24600 bp 25200 bp 25800 bp 26400 bp 27000 bp 27600 bp

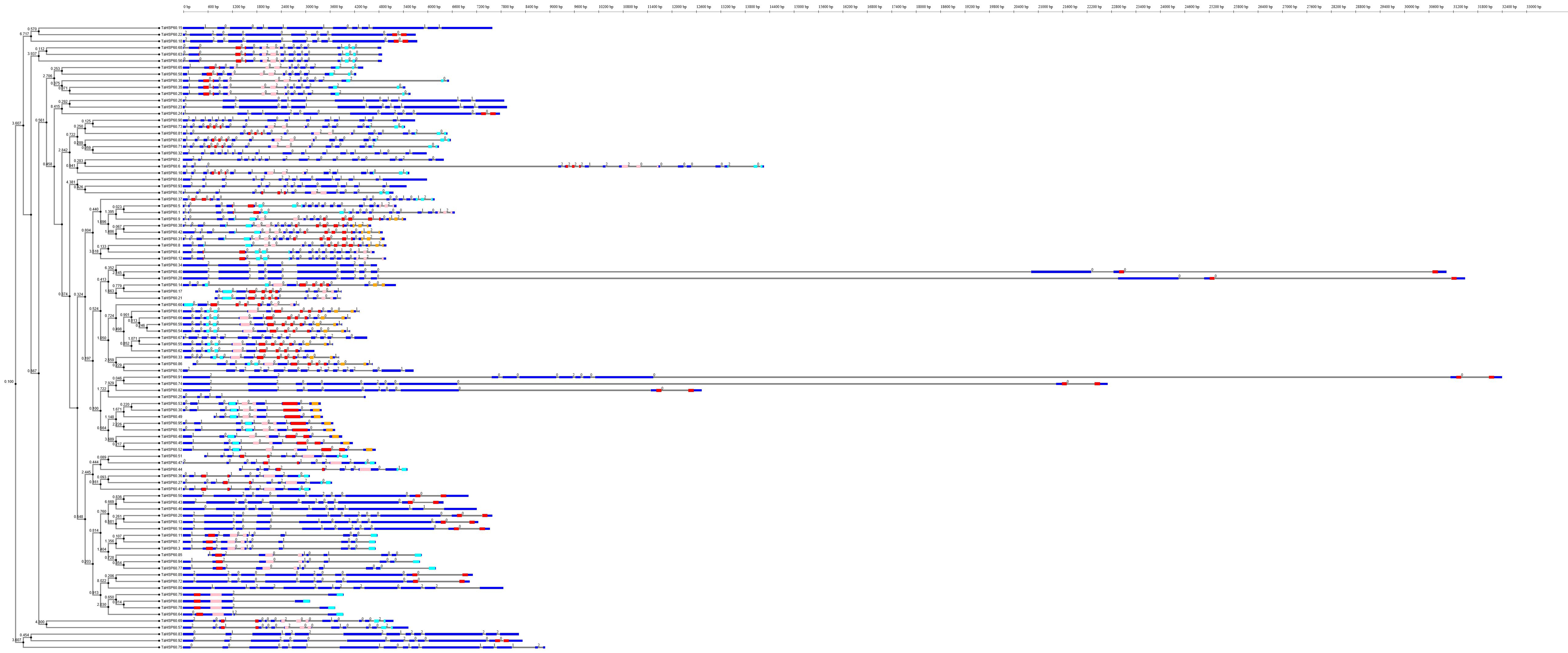
0 bp
600 bp
1200 bp
1800 bp
2400 bp
3000 bp
3600 bp
4200 bp
4800 bp
5400 bp
6000 bp
6600 bp
7200 bp
7800 bp
8400 bp
9000 bp
9600 bp
10200 bp
10800 bp
11400 bp
12000 bp
12600 bp
13200 bp
13800 bp
14400 bp
15000 bp
15600 bp
16200 bp
16800 bp
17400 bp
18000 bp
18600 bp
19200 bp
19800 bp
20400 bp
21000 bp
21600 bp
22200 bp
22800 bp
23400 bp
24000 bp
24600 bp
25200 bp
25800 bp
26400 bp
27000 bp
27600 bp
28200 bp
28800 bp
29400 bp
30000 bp
30600 bp
31200 bp
31800 bp
32400 bp
33000 bp
TaHSP60.15
TaHSP60.22
TaHSP60.18
TaHSP60.68
TaHSP60.63
TaHSP60.56
TaHSP60.65
TaHSP60.58
TaHSP60.39
TaHSP60.35
TaHSP60.29
TaHSP60.26
TaHSP60.23
TaHSP60.24
TaHSP60.90
TaHSP60.73
TaHSP60.81
TaHSP60.87
TaHSP60.71
TaHSP60.32
TaHSP60.2
TaHSP60.6
TaHSP60.10
TaHSP60.84
TaHSP60.93
TaHSP60.76
TaHSP60.37
TaHSP60.5
TaHSP60.1
TaHSP60.9
TaHSP60.38
TaHSP60.42
TaHSP60.31
TaHSP60.8
TaHSP60.4
TaHSP60.12
TaHSP60.34
TaHSP60.40
TaHSP60.28
TaHSP60.14
TaHSP60.17
TaHSP60.21
TaHSP60.60
TaHSP60.61
TaHSP60.66
TaHSP60.59
TaHSP60.54
TaHSP60.67
TaHSP60.55
TaHSP60.62
TaHSP60.33
TaHSP60.86
TaHSP60.70
TaHSP60.91
TaHSP60.74
TaHSP60.82
TaHSP60.25
TaHSP60.53
TaHSP60.30
TaHSP60.49
TaHSP60.95
TaHSP60.19
TaHSP60.48
TaHSP60.45
TaHSP60.52
TaHSP60.51
TaHSP60.47
TaHSP60.44
TaHSP60.36
TaHSP60.27
TaHSP60.41
TaHSP60.50
TaHSP60.43
TaHSP60.46
TaHSP60.20
TaHSP60.13
TaHSP60.16
TaHSP60.11
TaHSP60.7
TaHSP60.3
TaHSP60.85
TaHSP60.94
TaHSP60.77
TaHSP60.89
TaHSP60.72
TaHSP60.80
TaHSP60.79
TaHSP60.88
TaHSP60.78
TaHSP60.64
TaHSP60.69
TaHSP60.57
TaHSP60.83
TaHSP60.92
TaHSP60.75

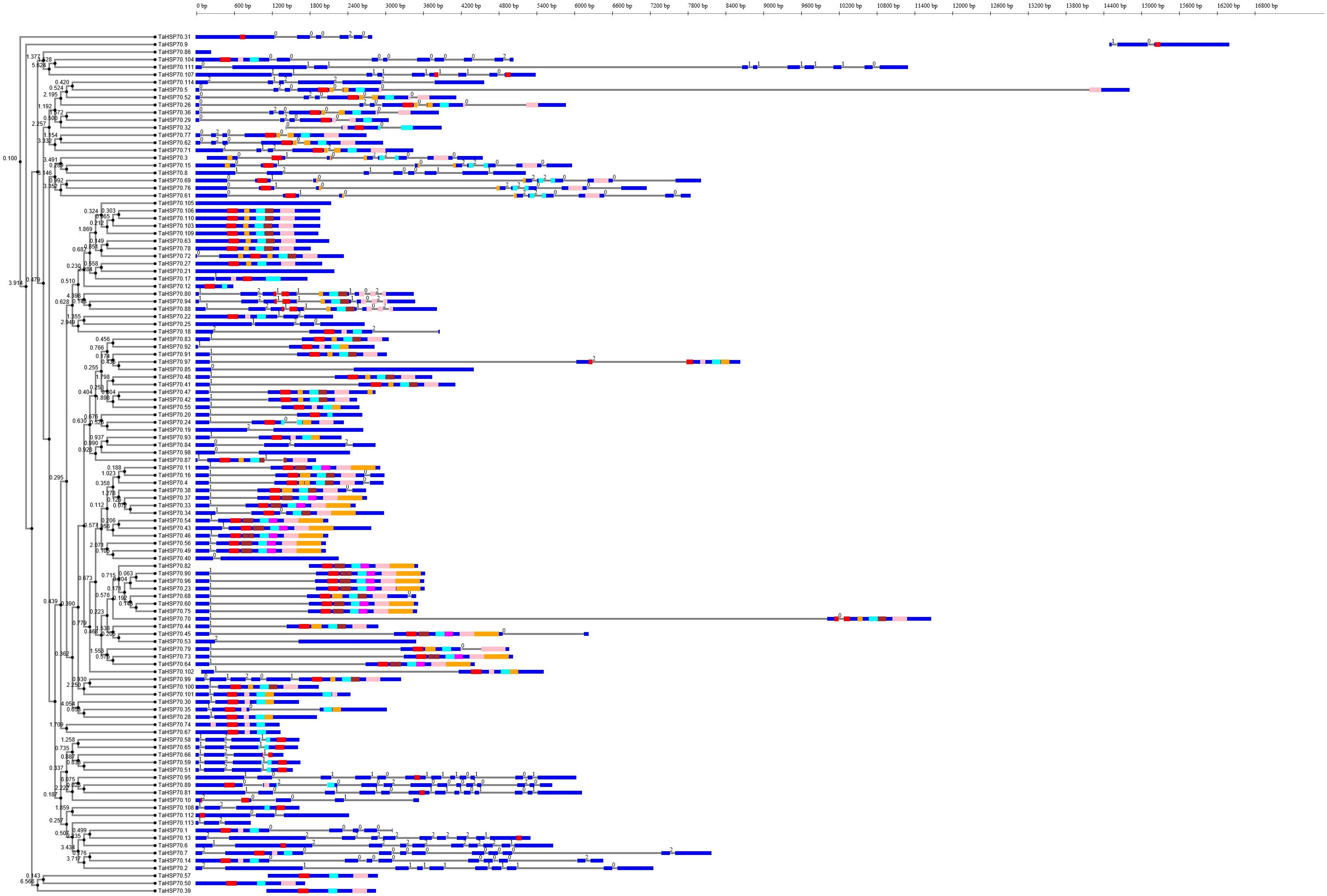

0 bp
600 bp
1200 bp
1800 bp
2400 bp
3000 bp
3600 bp
4200 bp
4800 bp
5400 bp
6000 bp
6600 bp
7200 bp
7800 bp
8400 bp
9000 bp
9600 bp
10200 bp
10800 bp
11400 bp
12000 bp
12600 bp
13200 bp
13800 bp
14400 bp
15000 bp
15600 bp
16200 bp
16800 bp
TaHSP70.31
TaHSP70.9
TaHSP70.86
TaHSP70.104
TaHSP70.111
TaHSP70.107
TaHSP70.114
TaHSP70.5
TaHSP70.52
TaHSP70.26
TaHSP70.36
TaHSP70.29
TaHSP70.32
TaHSP70.77
TaHSP70.62
TaHSP70.71
TaHSP70.3
TaHSP70.15
TaHSP70.8
TaHSP70.69
TaHSP70.76
TaHSP70.61
TaHSP70.105
TaHSP70.106
TaHSP70.110
TaHSP70.103
TaHSP70.109
TaHSP70.63
TaHSP70.78
TaHSP70.72
TaHSP70.27
TaHSP70.21
TaHSP70.17
TaHSP70.12
TaHSP70.80
TaHSP70.94
TaHSP70.88
TaHSP70.22
TaHSP70.25
TaHSP70.18
TaHSP70.83
TaHSP70.92
TaHSP70.91
TaHSP70.97
TaHSP70.85
TaHSP70.48
TaHSP70.41
TaHSP70.47
TaHSP70.42
TaHSP70.55
TaHSP70.20
TaHSP70.24
TaHSP70.19
TaHSP70.93
TaHSP70.84
TaHSP70.98
TaHSP70.87
TaHSP70.11
TaHSP70.16
TaHSP70.4
TaHSP70.38
TaHSP70.37
TaHSP70.33
TaHSP70.34
TaHSP70.54
TaHSP70.43
TaHSP70.46
TaHSP70.56
TaHSP70.49
TaHSP70.40
TaHSP70.82
TaHSP70.90
TaHSP70.96
TaHSP70.23
TaHSP70.68
TaHSP70.60
TaHSP70.75
TaHSP70.70
TaHSP70.44
TaHSP70.45
TaHSP70.53
TaHSP70.79
TaHSP70.73
TaHSP70.64
TaHSP70.102
TaHSP70.99
TaHSP70.100
TaHSP70.101
TaHSP70.30
TaHSP70.35
TaHSP70.28
TaHSP70.74
TaHSP70.67
TaHSP70.58
TaHSP70.65
TaHSP70.66
TaHSP70.59
TaHSP70.51
TaHSP70.95
TaHSP70.89
TaHSP70.81
TaHSP70.10
TaHSP70.108
TaHSP70.112
TaHSP70.113
TaHSP70.1
TaHSP70.13
TaHSP70.6
TaHSP70.7
TaHSP70.14
TaHSP70.2
TaHSP70.57
TaHSP70.50
TaHSP70.39

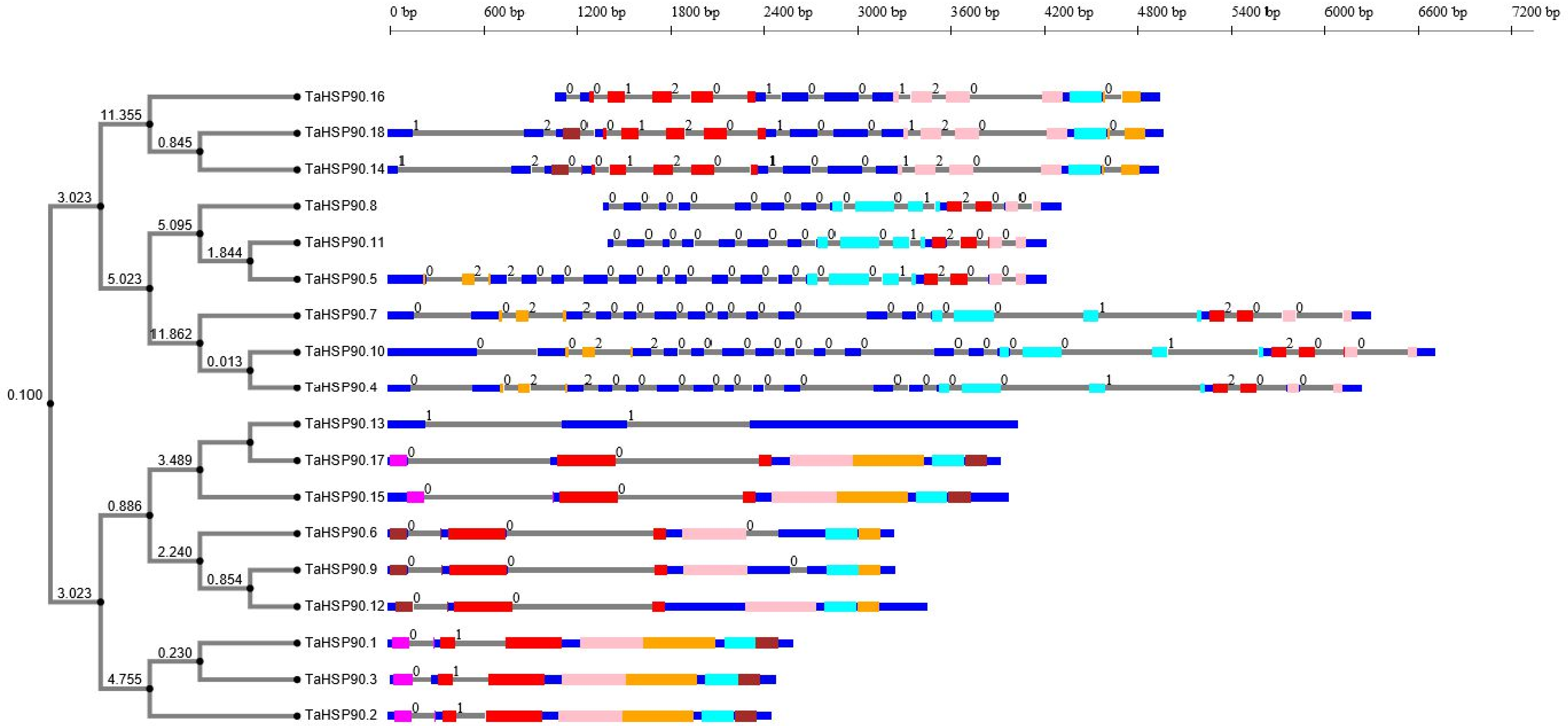

0 bp
600 bp
1200 bp
1800 bp
2400 bp
3000 bp
3600 bp
4200 bp
4800 bp
5400 bp
6000 bp
6600 bp
7200 bp
TaHSP90.16
TaHSP90.18
TaHSP90.14
TaHSP90.8
TaHSP90.11
TaHSP90.5
TaHSP90.7
TaHSP90.10
TaHSP90.4
TaHSP90.13
TaHSP90.17
TaHSP90.15
TaHSP90.6
TaHSP90.9
TaHSP90.12
TaHSP90.1
TaHSP90.3
TaHSP90.2
11.355
0.845
3.023
5.095
1.844
5.023
11.862
0.013
0.100
3.489
0.886
2.240
0.854
3.023
0.230
4.755

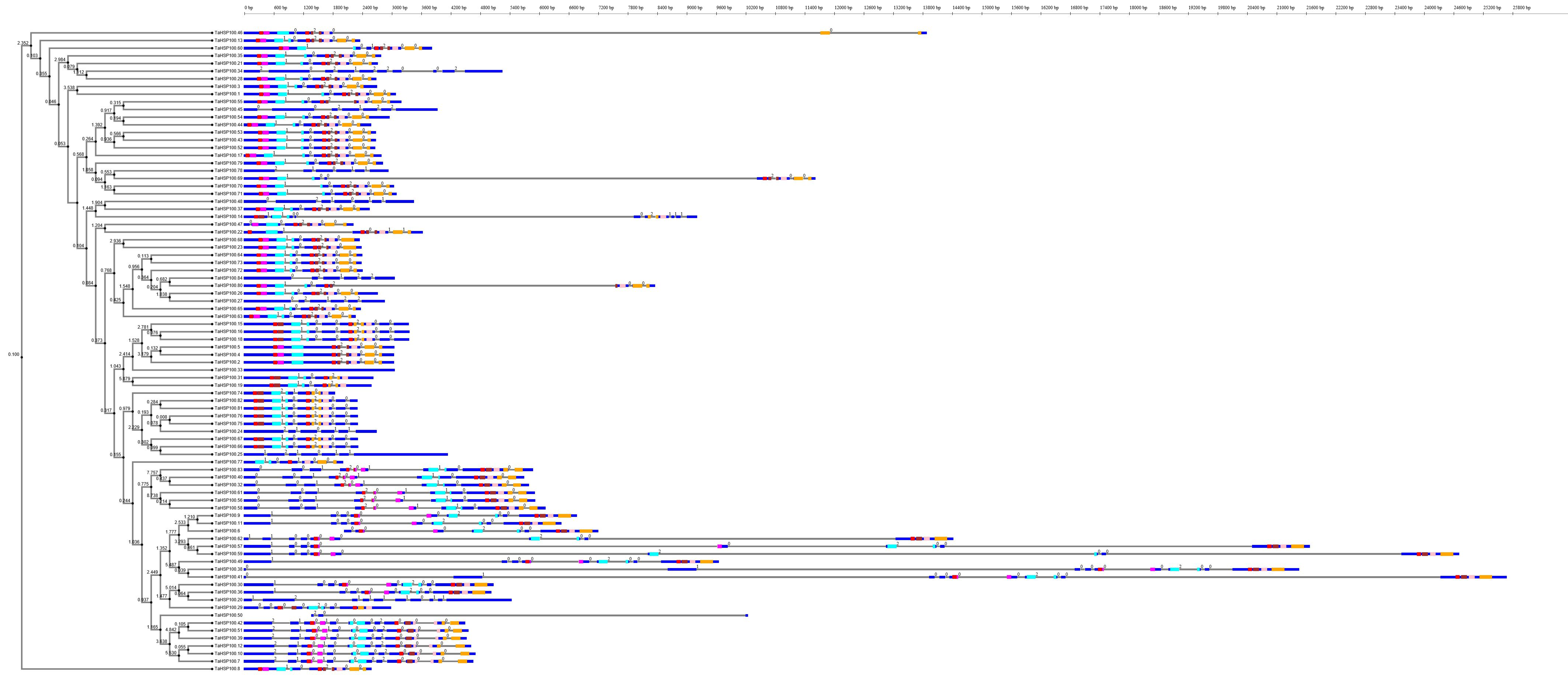
0 bp
600 bp
1200 bp
1800 bp
2400 bp
3000 bp
3600 bp
4200 bp
4800 bp
5400 bp
6000 bp
6600 bp
7200 bp
7800 bp
8400 bp
9000 bp
9600 bp
10200 bp
10800 bp
11400 bp
12000 bp
12600 bp
13200 bp
13800 bp
14400 bp
15000 bp
15600 bp
16200 bp
16800 bp
17400 bp
18000 bp
18600 bp
19200 bp
19800 bp
20400 bp
21000 bp
21600 bp
22200 bp
22800 bp
23400 bp
24000 bp
24600 bp
25200 bp
25800 bp
TaHSP100.46
TaHSP100.13
TaHSP100.60
TaHSP100.35
TaHSP100.21
TaHSP100.34
TaHSP100.28
TaHSP100.3
TaHSP100.1
TaHSP100.55
TaHSP100.45
TaHSP100.54
TaHSP100.44
TaHSP100.53
TaHSP100.43
TaHSP100.52
TaHSP100.17
TaHSP100.79
TaHSP100.78
TaHSP100.69
TaHSP100.70
TaHSP100.71
TaHSP100.48
TaHSP100.37
TaHSP100.14
TaHSP100.47
TaHSP100.22
TaHSP100.68
TaHSP100.23
TaHSP100.64
TaHSP100.73
TaHSP100.72
TaHSP100.84
TaHSP100.80
TaHSP100.26
TaHSP100.27
TaHSP100.65
TaHSP100.63
TaHSP100.15
TaHSP100.16
TaHSP100.18
TaHSP100.5
TaHSP100.4
TaHSP100.2
TaHSP100.33
TaHSP100.31
TaHSP100.19
TaHSP100.74
TaHSP100.82
TaHSP100.81
TaHSP100.76
TaHSP100.75
TaHSP100.24
TaHSP100.67
TaHSP100.66
TaHSP100.25
TaHSP100.77
TaHSP100.83
TaHSP100.40
TaHSP100.32
TaHSP100.61
TaHSP100.56
TaHSP100.58
TaHSP100.9
TaHSP100.11
TaHSP100.6
TaHSP100.62
TaHSP100.57
TaHSP100.59
TaHSP100.49
TaHSP100.38
TaHSP100.41
TaHSP100.30
TaHSP100.36
TaHSP100.20
TaHSP100.29
TaHSP100.50
TaHSP100.42
TaHSP100.51
TaHSP100.39
TaHSP100.12
TaHSP100.10
TaHSP100.7
TaHSP100.8

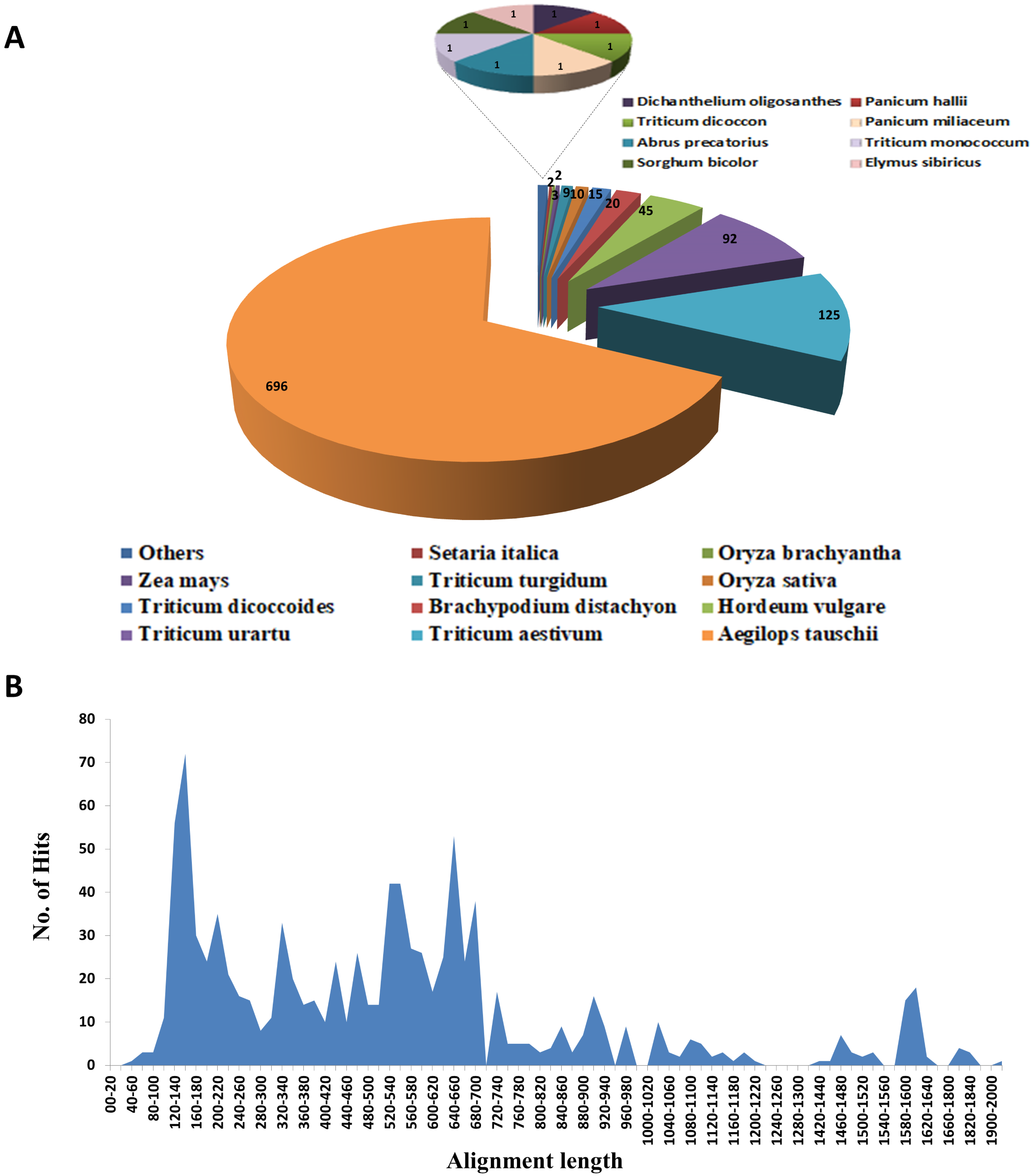

A
1
1
1
1
1
1
1
1
Dichanthelium oligosanthes
Panicum hallii
Triticum dicoccon
Panicum miliaceum
Abrus precatorius
Triticum monococcum
Sorghum bicolor
Elymus sibiricus
2
2
3
9
10
15
20
45
92
125
696
Others
Setaria italica
Oryza brachyantha
Zea mays
Triticum turgidum
Oryza sativa
Triticum dicoccoides
Brachypodium distachyon
Hordeum vulgare
Triticum urartu
Triticum aestivum
Aegilops tauschii
B
80
70
60
50
40
30
20
10
0
No. of Hits
00-20
40-60
80-100
120-140
160-180
200-220
240-260
280-300
320-340
360-380
400-420
440-460
480-500
520-540
560-580
600-620
640-660
680-700
720-740
760-780
800-820
840-860
880-900
920-940
960-980
1000-1020
1040-1060
1080-1100
1120-1140
1160-1180
1200-1220
1240-1260
1280-1300
1420-1440
1460-1480
1500-1520
1540-1560
1580-1600
1620-1640
1660-1800
1820-1840
1900-2000
Alignment length

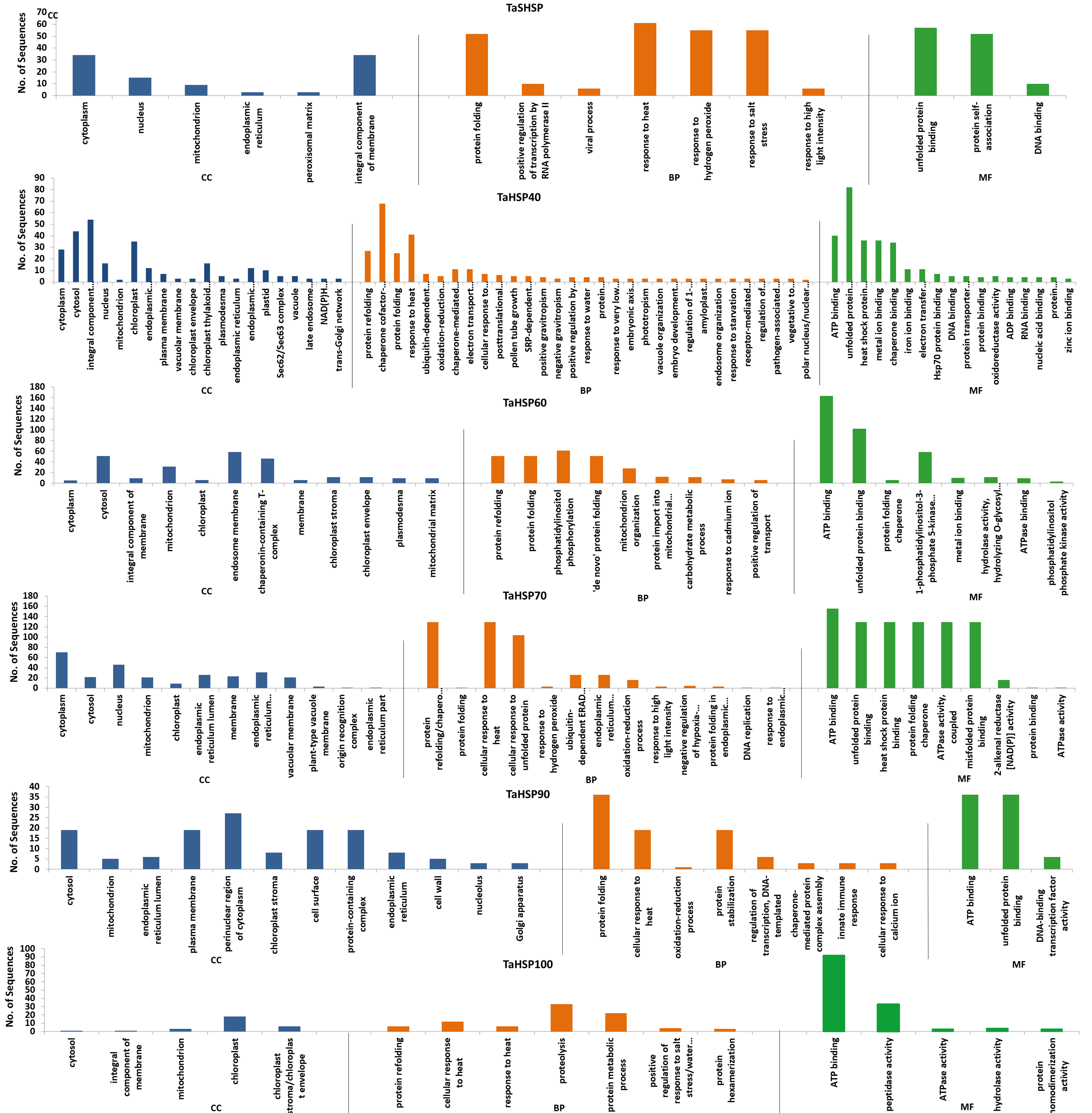

TaSHSP
No. of Sequences
CC
cytoplasm
nucleus
mitochondrion
endoplasmic reticulum
peroxisomal matrix
integral component of membrane
protein folding
positive regulation of transcription by RNA polymerase II
viral process
response to heat
response to hydrogen peroxide
response to salt stress
response to high light intensity
unfolded protein binding
protein self-association
DNA binding
CC
BP
MF
TaHSP40
No. of Sequences
cytoplasm
cytosol
integral component...
nucleus
mitochondrion
chloroplast
endoplasmic...
plasma membrane
vacuolar membrane
chloroplast envelope
chloroplast thylakoid...
plasmodesma
endoplasmic reticulum
endoplasmic...
plastid
Sec62/Sec63 complex
vacuole
late endosome...
NAD(P)H...
trans-Golgi network
protein refolding
chaperone cofactor-...
protein folding
response to heat
ubiquitin-dependent...
oxidation-reduction...
chaperone-mediated...
electron transport...
cellular response to...
posttranslational...
pollen tube growth
SRP-dependent...
positive gravitropism
negative gravitropism
positive regulation by...
response to water
protein...
response to very low...
embryonic axis...
phototropism
vacuole organization
embryo development...
regulation of 1-...
amyloplast...
endosome organization
response to starvation
receptor-mediated...
regulation of...
pathogen-associated...
vegetative to...
polar nucleus/nuclear...
ATP binding
unfolded protein...
heat shock protein...
metal ion binding
chaperone binding
iron ion binding
electron transfer...
Hsp70 protein binding
DNA binding
protein transporter...
protein binding
oxidoreductase activity
ADP binding
RNA binding
nucleic acid binding
protein...
zinc ion binding
CC
BP
MF
TaHSP60
No. of Sequences
cytoplasm
cytosol
integral component of membrane
mitochondrion
chloroplast
endosome membrane
chaperonin-containing T-complex
membrane
chloroplast stroma
chloroplast envelope
plasmodesma
mitochondrial matrix
protein refolding
protein folding
phosphatidylinositol phosphorylation
'de novo' protein folding
mitochondrion organization
protein import into mitochondrial...
carbohydrate metabolic process
response to cadmium ion
positive regulation of transport
ATP binding
unfolded protein binding
protein folding chaperone
1-phosphatidylinositol-3-phosphate 5-kinase...
metal ion binding
hydrolase activity, hydrolyzing O-glycosyl...
ATPase binding
phosphatidylinositol phosphate kinase activity
CC
BP
MF
TaHSP70
No. of Sequences
cytoplasm
cytosol
nucleus
mitochondrion
chloroplast
endoplasmic reticulum lumen
membrane
endoplasmic reticulum...
vacuolar membrane
plant-type vacuole membrane
origin recognition complex
endoplasmic reticulum part
protein refolding/chapero...
protein folding
cellular response to heat
cellular response to unfolded protein
response to hydrogen peroxide
ubiquitin-dependent ERAD...
endoplasmic reticulum...
oxidation-reduction process
response to high light intensity
negative regulation of hypoxia-...
protein folding in endoplasmic...
DNA replication
response to endoplasmic...
ATP binding
unfolded protein binding
heat shock protein binding
protein folding chaperone
ATPase activity, coupled
misfolded protein binding
2-alkenal reductase [NAD(P)] activity
protein binding
ATPase activity
CC
BP
MF
TaHSP90
No. of Sequences
cytosol
mitochondrion
endoplasmic reticulum lumen
plasma membrane
perinuclear region of cytoplasm
chloroplast stroma
cell surface
protein-containing complex
endoplasmic reticulum
cell wall
nucleolus
Golgi apparatus
protein folding
cellular response to heat
oxidation-reduction process
protein stabilization
regulation of transcription, DNA-templated
chaperone-mediated protein complex assembly
innate immune response
cellular response to calcium ion
ATP binding
unfolded protein binding
DNA-binding transcription factor activity
CC
BP
MF
TaHSP100
No. of Sequences
cytosol
integral component of membrane
mitochondrion
chloroplast
chloroplast stroma/chloroplast envelope
protein refolding
cellular response to heat
response to heat
proteolysis
protein metabolic process
positive regulation of response to salt stress/water...
protein hexamerization
ATP binding
peptidase activity
ATPase activity
hydrolase activity
protein homodimerization activity
CC
BP
MF

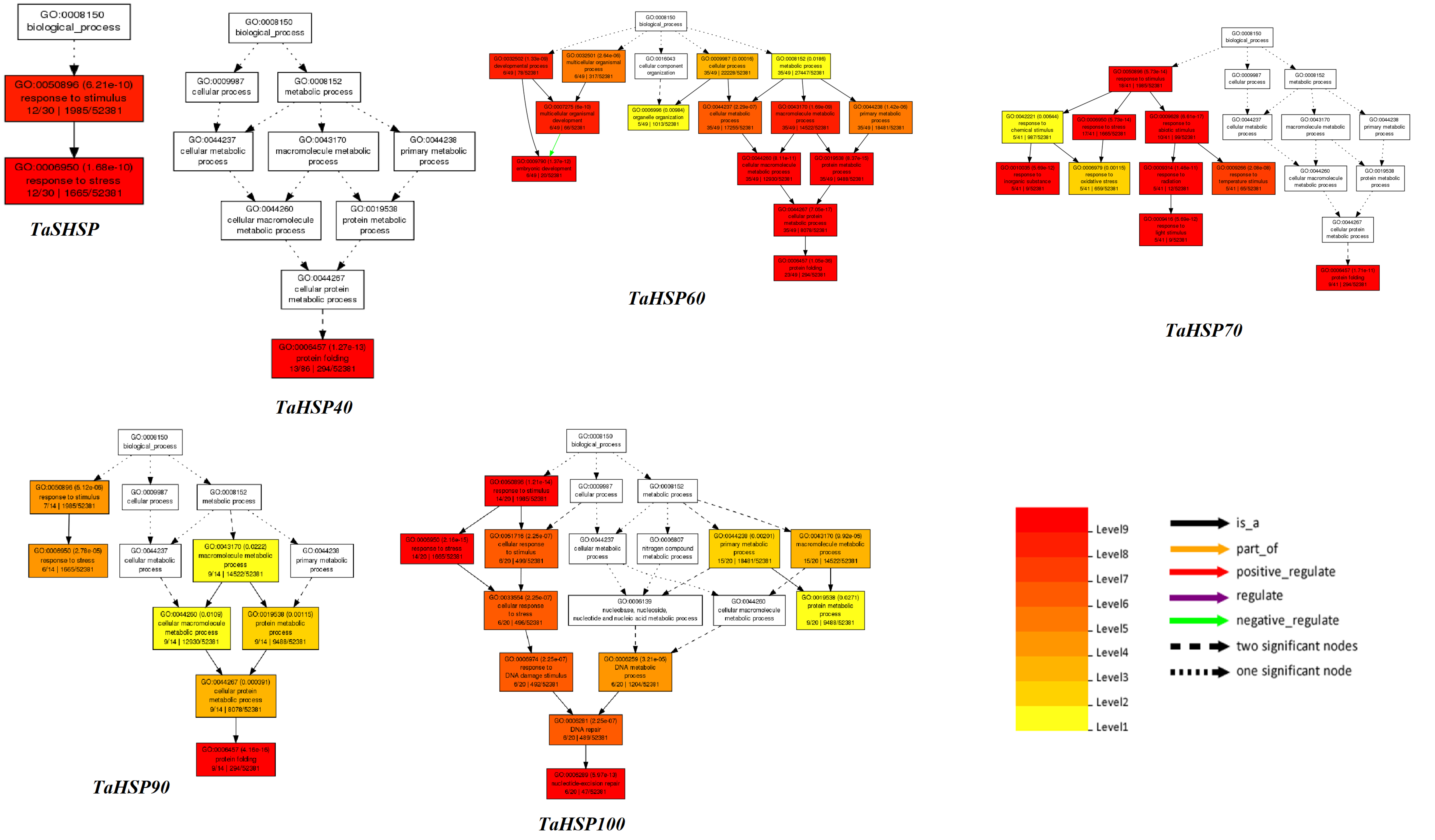

GO:0008150
biological_process
GO:0050896 (6.21e-10)
response to stimulus
12/30 | 1985/52381
GO:0006950 (1.68e-10)
response to stress
12/30 | 1665/52381
TaSHSP
GO:0008150
biological_process
GO:0009987
cellular process
GO:0008152
metabolic process
GO:0044237
cellular metabolic process
GO:0043170
macromolecule metabolic process
GO:0044238
primary metabolic process
GO:0044260
cellular macromolecule metabolic process
GO:0019538
protein metabolic process
GO:0044267
cellular protein metabolic process
GO:0006457 (1.27e-13)
protein folding
13/86 | 294/52381
TaHSP40
GO:0008150
biological_process
GO:0032502 (1.33e-09)
developmental process
6/49 | 78/52381
GO:0032501 (2.64e-06)
multicellular organismal process
6/49 | 317/52381
GO:0016043
cellular component organization
GO:0009987 (0.00016)
cellular process
35/49 | 22228/52381
GO:0008152 (0.0186)
metabolic process
35/49 | 27447/52381
GO:0007275 (6e-10)
multicellular organismal development
6/49 | 66/52381
GO:0006996 (0.00984)
organelle organization
5/49 | 1013/52381
GO:0044237 (2.29e-07)
cellular metabolic process
35/49 | 17255/52381
GO:0043170 (1.69e-09)
macromolecule metabolic process
35/49 | 14522/52381
GO:0044238 (1.42e-06)
primary metabolic process
35/49 | 18481/52381
GO:0009790 (1.37e-12)
embryonic development
6/49 | 20/52381
GO:0044260 (8.11e-11)
cellular macromolecule metabolic process
35/49 | 12930/52381
GO:0019538 (8.37e-15)
protein metabolic process
35/49 | 9488/52381
GO:0044267 (7.05e-17)
cellular protein metabolic process
35/49 | 8078/52381
GO:0006457 (1.05e-36)
protein folding
23/49 | 294/52381
TaHSP60
GO:0008150
biological_process
GO:0050896 (5.73e-14)
response to stimulus
18/41 | 1985/52381
GO:0009987
cellular process
GO:0008152
metabolic process
GO:0042221 (0.00644)
response to chemical stimulus
5/41 | 987/52381
GO:0006950 (5.73e-14)
response to stress
17/41 | 1665/52381
GO:0009628 (6.61e-17)
response to abiotic stimulus
10/41 | 99/52381
GO:0044237
cellular metabolic process
GO:0043170
macromolecule metabolic process
GO:0044238
primary metabolic process
GO:0010035 (5.69e-12)
response to inorganic substance
5/41 | 9/52381
GO:0006979 (0.00115)
response to oxidative stress
5/41 | 659/52381
GO:0009314 (1.46e-11)
response to radiation
5/41 | 12/52381
GO:0009266 (2.08e-08)
response to temperature stimulus
5/41 | 65/52381
GO:0044260
cellular macromolecule metabolic process
GO:0019538
protein metabolic process
GO:0009416 (5.69e-12)
response to light stimulus
5/41 | 9/52381
GO:0044267
cellular protein metabolic process
GO:0006457 (1.71e-11)
protein folding
9/41 | 294/52381
TaHSP70
GO:0008150
biological_process
GO:0050896 (5.12e-06)
response to stimulus
7/14 | 1985/52381
GO:0009987
cellular process
GO:0008152
metabolic process
GO:0006950 (2.78e-05)
response to stress
6/14 | 1665/52381
GO:0044237
cellular metabolic process
GO:0043170 (0.0222)
macromolecule metabolic process
9/14 | 14522/52381
GO:0044238
primary metabolic process
GO:0044260 (0.0109)
cellular macromolecule metabolic process
9/14 | 12930/52381
GO:0019538 (0.00115)
protein metabolic process
9/14 | 9488/52381
GO:0044267 (0.000391)
cellular protein metabolic process
9/14 | 8078/52381
GO:0006457 (4.16e-16)
protein folding
9/14 | 294/52381
TaHSP90
GO:0008150
biological_process
GO:0050896 (1.21e-14)
response to stimulus
14/20 | 1985/52381
GO:0009987
cellular process
GO:0008152
metabolic process
GO:0006950 (2.16e-15)
response to stress
14/20 | 1665/52381
GO:0051716 (2.25e-07)
cellular response to stimulus
6/20 | 499/52381
GO:0044237
cellular metabolic process
GO:0006807
nitrogen compound metabolic process
GO:0044238 (0.00201)
primary metabolic process
15/20 | 18481/52381
GO:0043170 (9.92e-05)
macromolecule metabolic process
15/20 | 14522/52381
GO:0033554 (2.25e-07)
cellular response to stress
6/20 | 496/52381
GO:0006139
nucleobase, nucleoside, nucleotide and nucleic acid metabolic process
GO:0044260
cellular macromolecule metabolic process
GO:0019538 (0.0271)
protein metabolic process
9/20 | 9488/52381
GO:0006974 (2.25e-07)
response to DNA damage stimulus
6/20 | 492/52381
GO:0006259 (3.21e-05)
DNA metabolic process
6/20 | 1204/52381
GO:0006281 (2.25e-07)
DNA repair
6/20 | 489/52381
GO:0006289 (5.97e-13)
nucleotide-excision repair
6/20 | 47/52381
TaHSP100
Level9
Level8
Level7
Level6
Level5
Level4
Level3
Level2
Level1
is_a
part_of
positive_regulate
regulate
negative_regulate
two significant nodes
one significant node

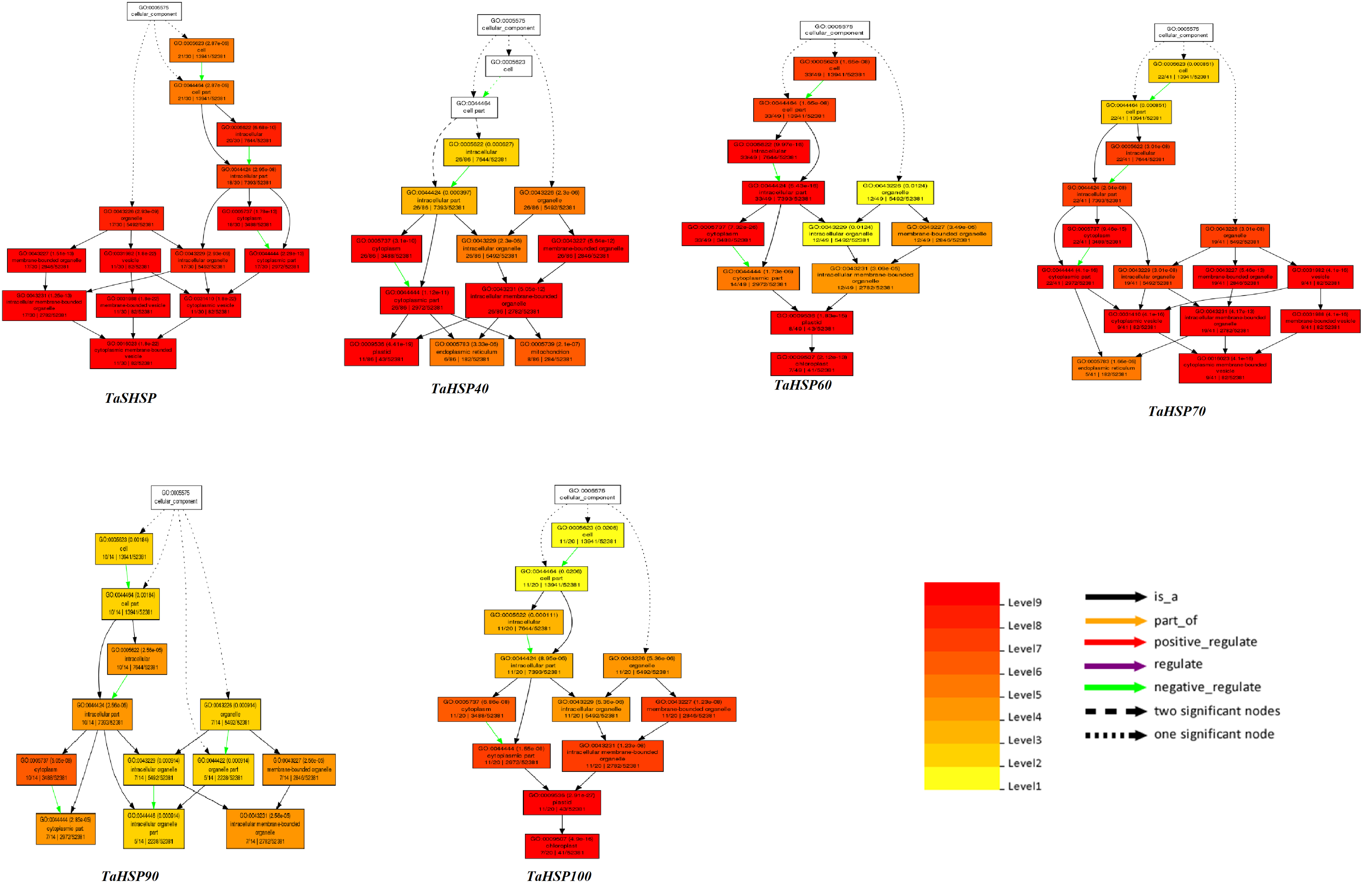

TaSHSP
TaHSP40
TaHSP60
TaHSP70
TaHSP90
TaHSP100
Level9
Level8
Level7
Level6
Level5
Level4
Level3
Level2
Level1
is_a
part_of
positive_regulate
regulate
negative_regulate
two significant nodes
one significant node

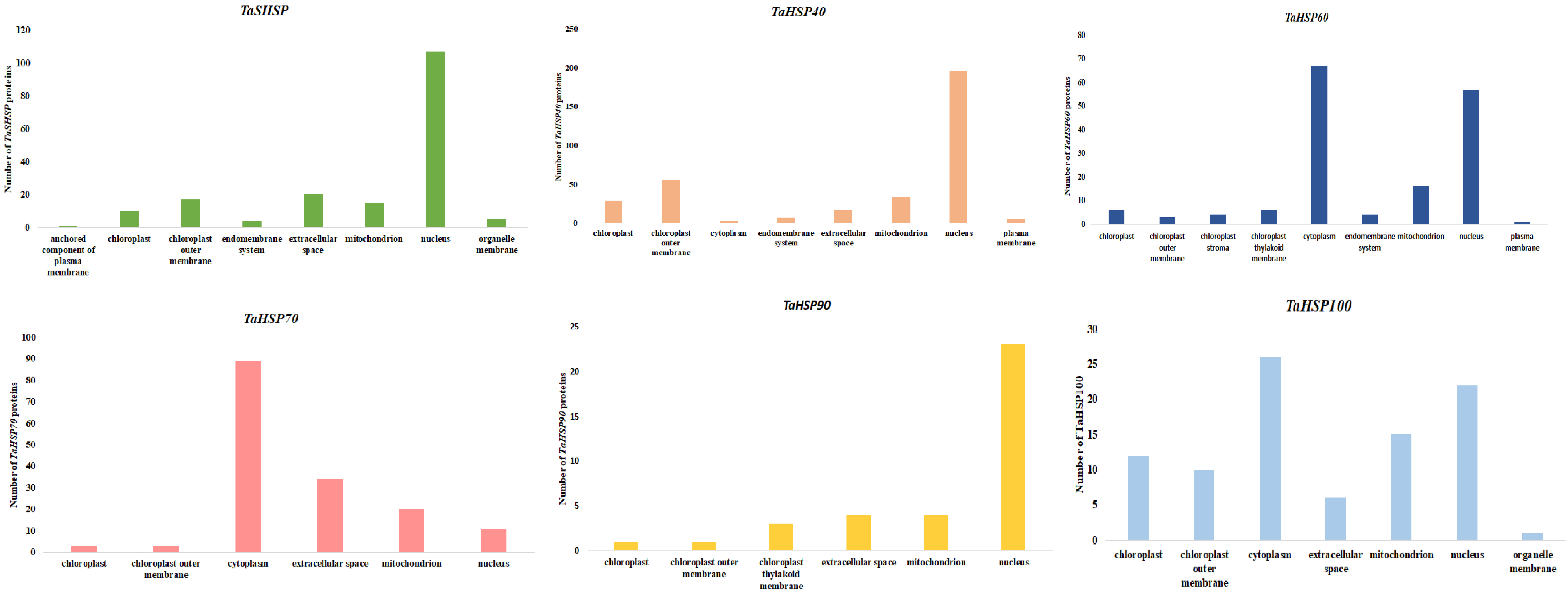

TaSHSP
Number of TaSHSP proteins
anchored component of plasma membrane
chloroplast
chloroplast outer membrane
endomembrane system
extracellular space
mitochondrion
nucleus
organelle membrane
TaHSP40
Number of TaHSP40 proteins
chloroplast
chloroplast outer membrane
cytoplasm
endomembrane system
extracellular space
mitochondrion
nucleus
plasma membrane
TaHSP60
Number of TaHSP60 proteins
chloroplast
chloroplast outer membrane
chloroplast stroma
chloroplast thylakoid membrane
cytoplasm
endomembrane system
mitochondrion
nucleus
plasma membrane
TaHSP70
Number of TaHSP70 proteins
chloroplast
chloroplast outer membrane
cytoplasm
extracellular space
mitochondrion
nucleus
TaHSP90
Number of TaHSP90 proteins
chloroplast
chloroplast outer membrane
chloroplast thylakoid membrane
extracellular space
mitochondrion
nucleus
TaHSP100
Number of TaHSP100
chloroplast
chloroplast outer membrane
cytoplasm
extracellular space
mitochondrion
nucleus
organelle membrane

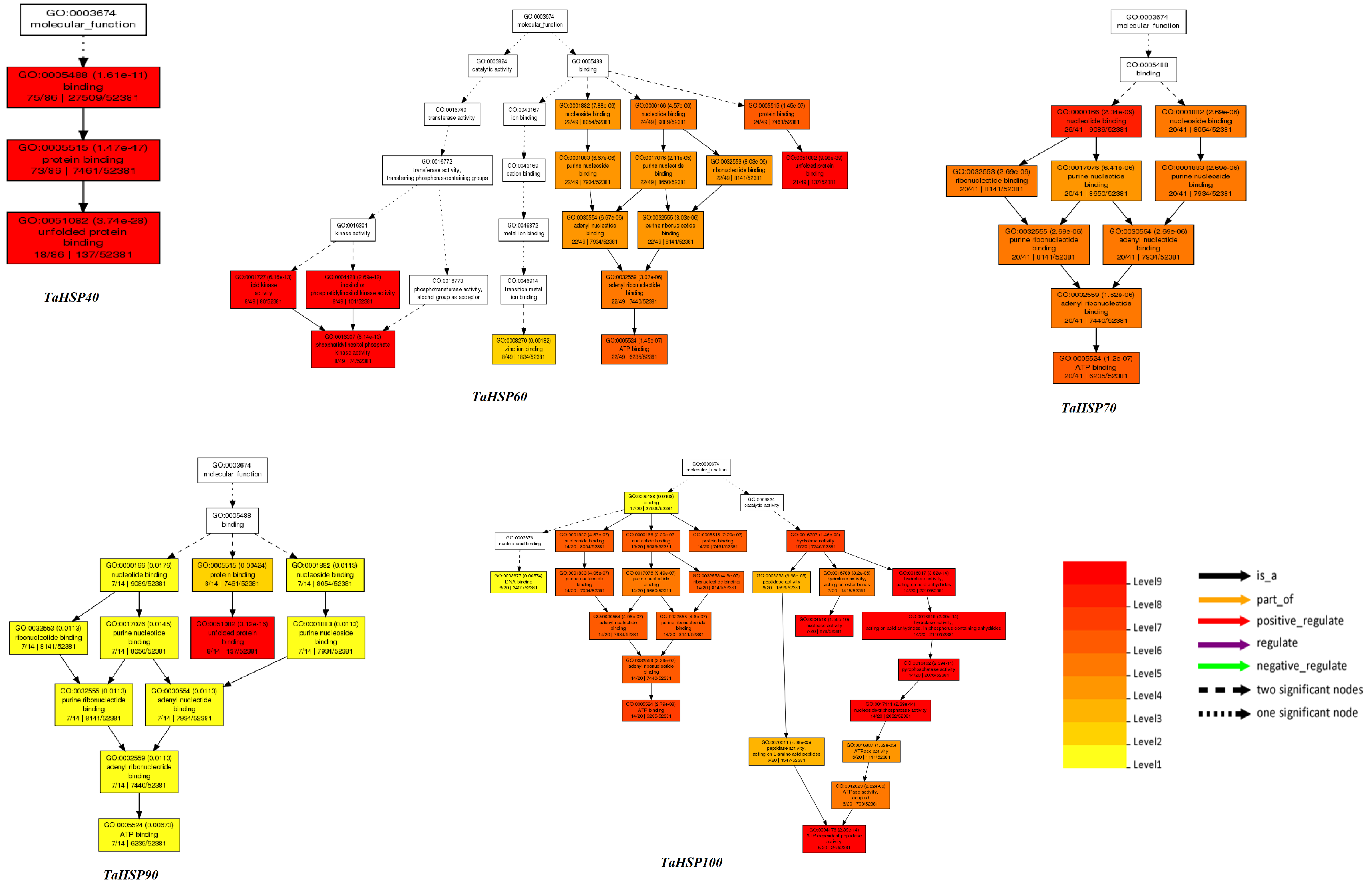

GO:0003674
molecular_function
GO:0005488 (1.61e-11)
binding
75/86 | 27509/52381
GO:0005515 (1.47e-47)
protein binding
73/86 | 7461/52381
GO:0051082 (3.74e-28)
unfolded protein
binding
18/86 | 137/52381
TaHSP40
TaHSP60
GO:0005488
binding
GO:0000166 (2.34e-09)
nucleotide binding
26/41 | 9089/52381
GO:0001882 (2.69e-06)
nucleoside binding
20/41 | 8054/52381
GO:0032553 (2.69e-06)
ribonucleotide binding
20/41 | 8141/52381
GO:0017076 (6.41e-06)
purine nucleotide
binding
20/41 | 8650/52381
GO:0001883 (2.69e-06)
purine nucleoside
binding
20/41 | 7934/52381
GO:0032555 (2.69e-06)
purine ribonucleotide
binding
20/41 | 8141/52381
GO:0030554 (2.69e-06)
adenyl nucleotide
binding
20/41 | 7934/52381
GO:0032559 (1.62e-06)
adenyl ribonucleotide
binding
20/41 | 7440/52381
GO:0005524 (1.2e-07)
ATP binding
20/41 | 6235/52381
TaHSP70
GO:0000166 (0.0176)
nucleotide binding
7/14 | 9089/52381
GO:0005515 (0.00424)
protein binding
8/14 | 7461/52381
GO:0001882 (0.0113)
nucleoside binding
7/14 | 8054/52381
GO:0032553 (0.0113)
ribonucleotide binding
7/14 | 8141/52381
GO:0017076 (0.0145)
purine nucleotide
binding
7/14 | 8650/52381
GO:0051082 (3.12e-16)
unfolded protein
binding
8/14 | 137/52381
GO:0001883 (0.0113)
purine nucleoside
binding
7/14 | 7934/52381
GO:0032555 (0.0113)
purine ribonucleotide
binding
7/14 | 8141/52381
GO:0030554 (0.0113)
adenyl nucleotide
binding
7/14 | 7934/52381
GO:0032559 (0.0113)
adenyl ribonucleotide
binding
7/14 | 7440/52381
GO:0005524 (0.00673)
ATP binding
7/14 | 6235/52381
TaHSP90
TaHSP100
Level9
Level8
Level7
Level6
Level5
Level4
Level3
Level2
Level1
is_a
part_of
positive_regulate
regulate
negative_regulate
two significant nodes
one significant node

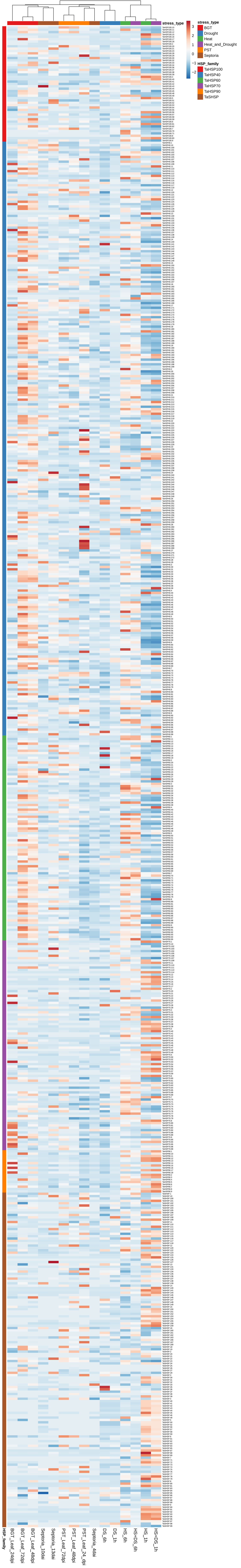

stress_type
3
2
1
0
−1
−2
stress_type
BGT
Drought
Heat
Heat_and_Drought
PST
Septoria
HSP_family
TaHSP100
TaHSP40
TaHSP60
TaHSP70
TaHSP90
TaSHSP
HSP_family
BGT_Leaf_24dpi
BGT_Leaf_72dpi
BGT_Leaf_48dpi
Septoria_10dai
Septoria_13dai
PST_Leaf_72dpi
PST_Leaf_48dpi
PST_Leaf_24_dpi
Septoria_4dai
DS_6h
DS_1h
HS_6h
HS+DS_6h
HS_1h
HS+DS_1h

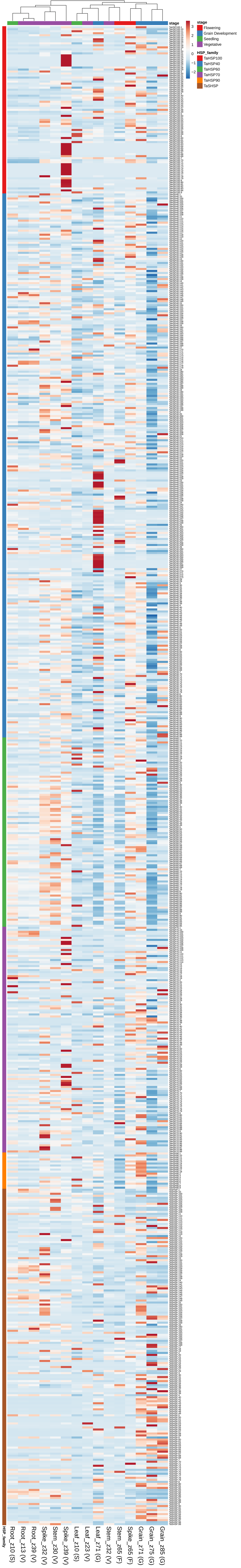
stage
3
2
1
0
−1
−2
stage
Flowering
Grain Development
Seedling
Vegetative
HSP_family
TaHSP100
TaHSP40
TaHSP60
TaHSP70
TaHSP90
TaSHSP
HSP_family
Root_z10 (S)
Root_z13 (V)
Root_z39 (V)
Spike_z32 (V)
Stem_z30 (V)
Spike_z39 (V)
Leaf_z10 (S)
Leaf_z23 (V)
Leaf_z71 (G)
Stem_z32 (V)
Stem_z65 (F)
Spike_z65 (F)
Grain_z71 (G)
Grain_z75 (G)
Grain_z85 (G)

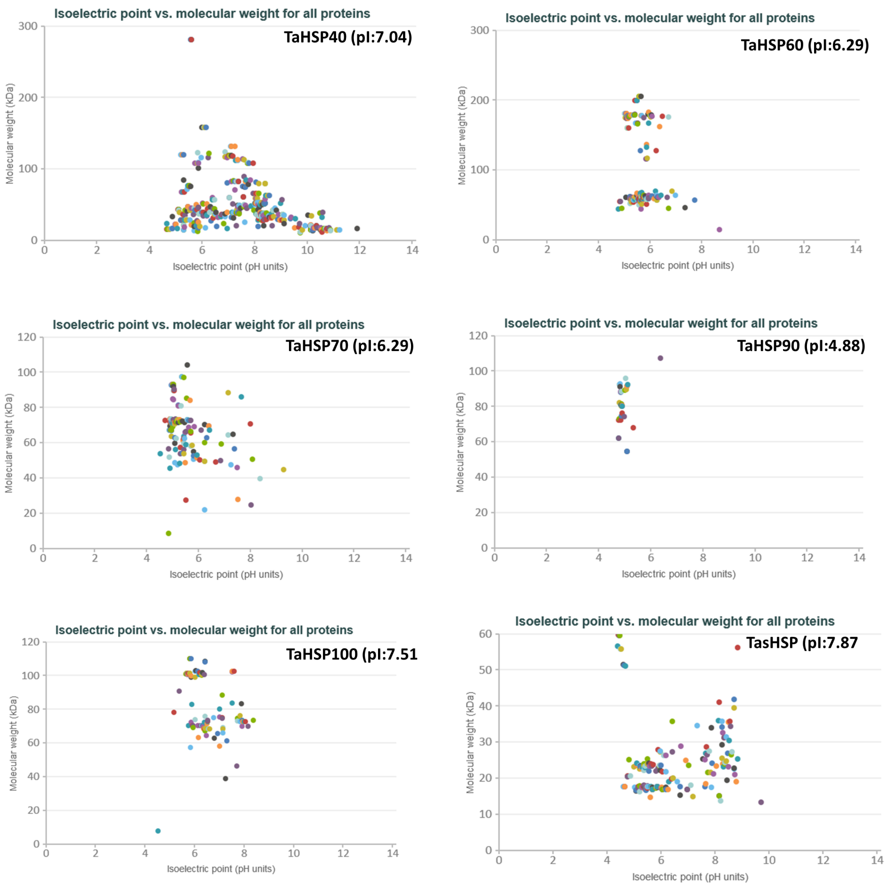
Isoelectric point vs. molecular weight for all proteins
TaHSP40 (pI:7.04)
Isoelectric point vs. molecular weight for all proteins
TaHSP60 (pI:6.29)
Isoelectric point vs. molecular weight for all proteins
TaHSP70 (pI:6.29)
Isoelectric point vs. molecular weight for all proteins
TaHSP90 (pI:4.88)
Isoelectric point vs. molecular weight for all proteins
TaHSP100 (pI:7.51
Isoelectric point vs. molecular weight for all proteins
TasHSP (pI:7.87
Molecular weight (kDa)
Isoelectric point (pH units)

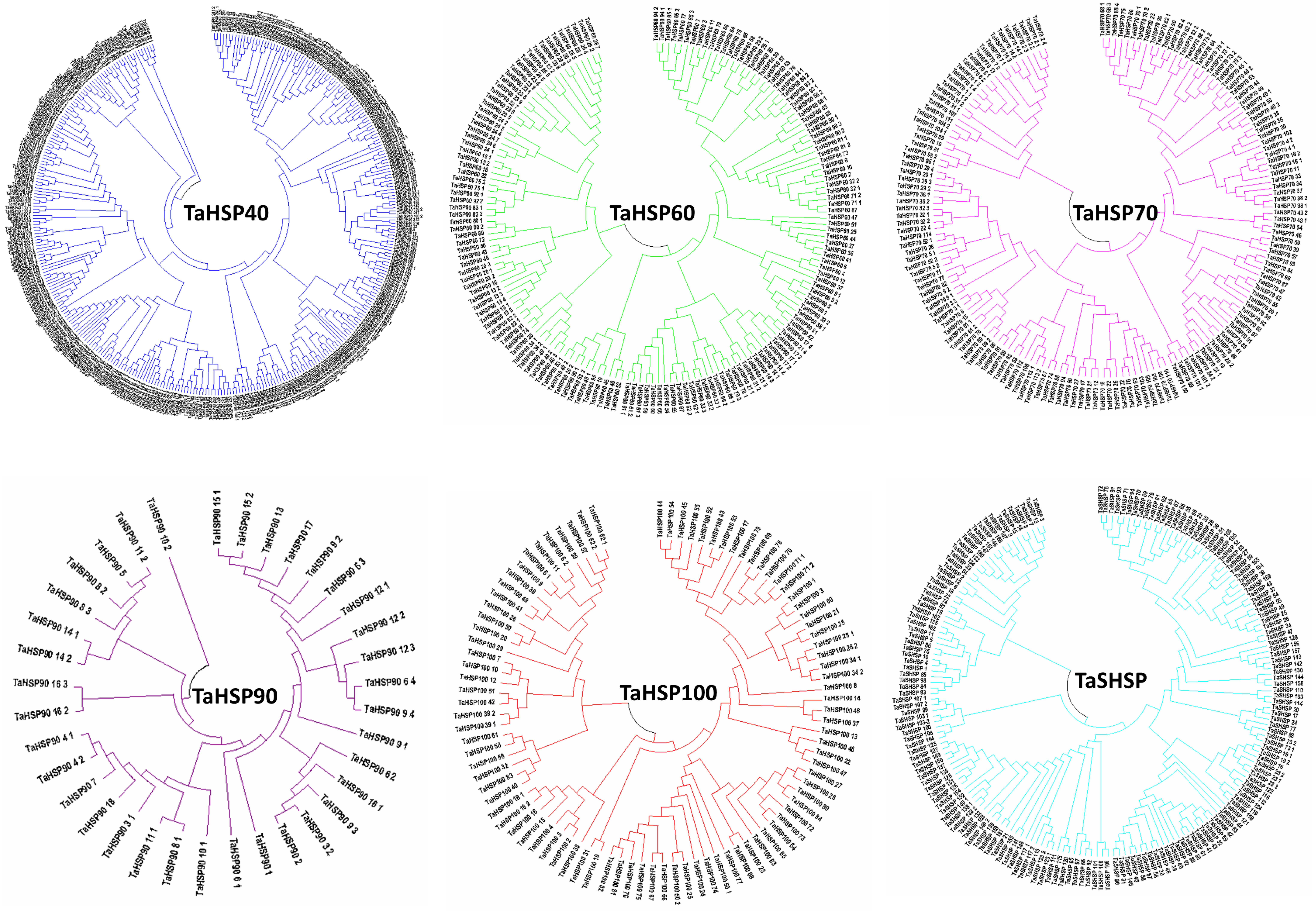
TaHSP40
TaHSP60
TaHSP70
TaHSP90
TaHSP100
TaSHSP

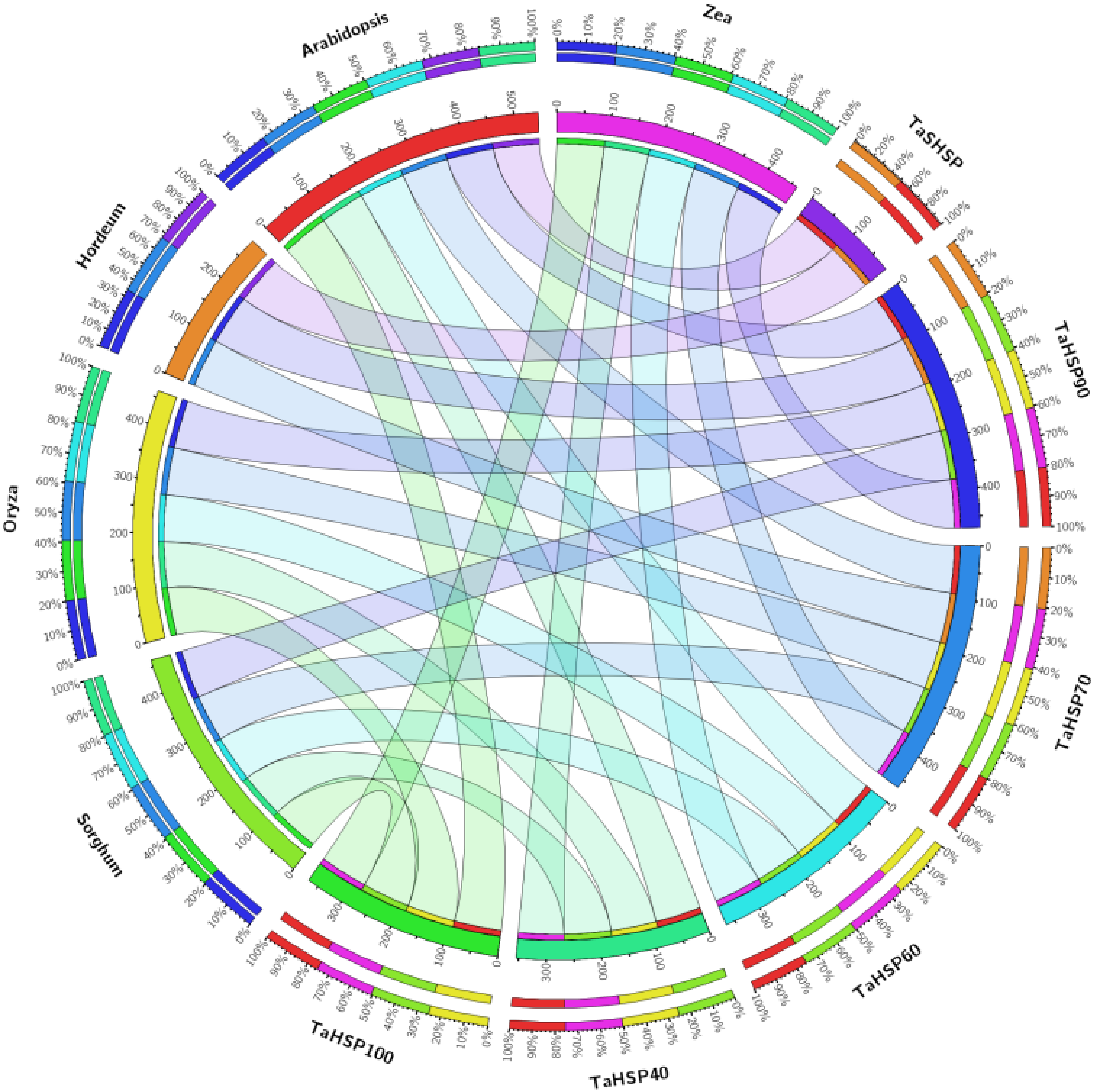
Zea
TaSHSP
TaHSP90
TaHSP70
TaHSP60
TaHSP40
TaHSP100
Sorghum
Oryza
Hordeum
Arabidopsis

Supplementary Data

Figure S1: Conserved Motifs of *TaHSP*s elucidated by MEME. Colored boxes representing different conserved motifs having different sequences and sizes.

Figure S2: Gene structure of all the 753 *TaHSP* genes, prepared using GSDraw

Figure S3: Blast2GO statistics based on BLAST search against the NR database

A: BLAST top-hit species distribution, **B**: Sequence similarity distribution

Figure S4: Bar graph representing the number of sequences of different subfamilies of *TaHSPs* distributed among various Cellular component (CC) Biological Process (BP) and Molecular Function (MF) depicted by blue, orange and green color.

Figure S5: Gene ontology term distribution in different HSP sub-families predicted using BLAST2GO in Biological Process.

Figure S6: Gene ontology term distribution in different HSP sub-families predicted using BLAST2GO in Cellular component

Figure S7: Sub-cellular localization predicted using BUSCO

Figure S8: Gene ontology term distribution in different HSP sub-families predicted using BLAST2GO in Molecular function.

Figure S9: Heatmaps representing the expression of all 753 *TaHSP* genes in various developmental stages. FPKM values were directly used to create the heatmaps.

Figure S10: Heatmaps representing the expression of all the 753 *TaHSP* genes in various stress conditions. FPKM values were directly used to create the heatmaps. Pst: *Puccinia striiformis*, Bgt: *Blumeria graminis*, dpi: days post inoculation

Figure S11: Isoelectric point vs. Molecular weight (kDa) plots of different *TaHSPs* subfamily.

Figure S12: Phylogenetic relationship within members of the different HSP sub-families in wheat, inferred using maximum likelihood method. A: *TaHSP40*; B: *TaHSP60*; C: *TaHSP70*; D: *TaHSP90*; E: *TaHSP100*, F: *TaSHSP*

Figure S13: Syntenic relationship of *TaHSP*s orthologous with its different monocots and dicots viz., *O. sativa, H. vulgure, S. bicolor, Z. mays* and *A. thaliana.*
